# Spanish Paediatric Haematology and Oncology Survival Results and Trends, 1999–2022

**DOI:** 10.3390/cancers18030362

**Published:** 2026-01-23

**Authors:** Pau Alfonso-Comos, Álvaro Briz-Redón, José Luis Dapena Díaz, Susana Rives, José María Fernández Navarro, Jaime Verdú-Amorós, Adela Cañete, RETI-SEHOP Survival Working Group

**Affiliations:** 1 Spanish Registry of Childhood Tumours (RETI-SEHOP), Faculty of Medicine, University of Valencia, 46010 Valencia, Spain; 2 Department of Statistics and Operations Research, Faculty of Mathematics, University of Valencia, 46100 Valencia, Spain; 3 Leukaemia and Lymphoma Department, Paediatric Cancer Centre Barcelona (PCCB), Sant Joan de Déu Hospital, 08950 Barcelona, Spain; 4 Paediatric Haematology and Oncology Unit, La Fe University and Polytechnic Hospital, 46026 Valencia, Spain; 5 Department of Paediatric Haematology and Oncology, Biomedical Research Institute INCLIVA, University Clinical Hospital, 46010 Valencia, Spain; 6 Centro de Investigación Biomédica en Red de Cáncer (CIBERONC), 28029 Madrid, Spain

**Keywords:** childhood cancer, survival, cancer registries, trends, epidemiology

## Abstract

**Background**: Childhood cancer is the leading cause of natural death among children in high-income countries, despite treatment improvements. The Spanish Registry of Childhood Tumours (RETI-SEHOP) systematically records all cases treated within the network of SEHOP units. Using RETI-SEHOP data, we evaluated survival trends to assess progress in patient care, both overall and by tumour. **Methods**: A total of 20,534 childhood cancer cases (0–14 years) were recorded across the period 1999–2021. The 1-, 3-, and 5-year overall survival (OS) were estimated using the Kaplan–Meier method, applying the cohort approach for 1999–2018 and the period approach for 2019–2022. OS by age and sex was analysed in the recent 2009–2018 incidence cohort. Age-adjusted OS time trends were examined using joinpoint Cox models for 1999–2022. **Results**: For all tumours combined, 5-year OS increased from 75.4% to 84.6% between 1999–2003 and 2019–2022. While positive trends were identified for all haematological malignancies examined, a more varied scenario was evident for solid tumours, as ependymomas improved fastest (1.51 points annually), and sarcomas, except for rhabdomyosarcoma, remained stagnant. **Conclusions**: Our results reflect a period characterised by a combination of new therapeutic developments, improved diagnostics, and more refined risk stratification, which has ultimately led to a reduction in disease-related mortality.

## Simple Summary

Childhood cancer survival is a key indicator for evaluating therapeutic efficacy and the functioning of health care systems. This study uses data from 20,534 childhood cancer cases (0–14 years) treated at Spanish paediatric haematology and oncology units from 1999 to 2021. We report retrospective nationwide survival results for major diagnostic groups and clinically relevant subtypes. To provide the most up-to-date picture, we used the period approach to estimate survival for recent years (2019–2022), which predicts the outcomes that will eventually be observed once follow-up is complete. We analysed survival trends to evaluate progress for each tumour, identifying potential change points. Our findings show that 5-year overall survival in Spain for the period 2019–2022 was 84.6%, with notable improvements in haematological malignancies, ependymomas, medulloblastomas, neuroblastomas, and rhabdomyosarcomas. These results aim to maximise comparability across registries and provide essential benchmarks for clinicians, researchers, and policymakers.

## 1. Introduction

Childhood cancer is a heterogeneous group of rare neoplasms that jointly account for fewer than 1% of all cancer cases in Europe. Nonetheless, childhood cancer remains the leading cause of natural death among children and adolescents in high-income countries (HIC), despite substantial reductions in mortality [1]. In Southern Europe, the age-standardised incidence of cancer in children (<15 years) was estimated at 170.9 cases per million across the period of 2001–2010 [2]. Currently, approximately 1000 cancer cases <15 years are expected annually in Spain, and more than 96% are diagnosed and managed at paediatric oncology or haematology units [3].

In Europe, 5-year survival among children with cancer exceeded 80% roughly a decade ago [4] and may now be approaching 85% in highly developed European countries [5,6,7]. These improvements have largely resulted from the co-ordinated efforts of international collaborative groups and consortia, leading to refined risk stratification, the optimisation of chemotherapy regimens, the development of immunotherapy, and the integration of molecular marker data [8,9]. While advances in surgical techniques have improved the safety and completeness of tumour resections [10,11,12], modern imaging technologies allow for earlier detection of metastatic disease [13]. In the context of high survival rates in HIC, childhood cancer care is increasingly focusing on preventing late effects and preserving organ function [14,15,16].

Cancer registries are essential instruments for documenting and monitoring the burden of cancer through the surveillance of incidence, mortality, survival, and prevalence [17]. In Spain, the Spanish Registry of Childhood Tumours (RETI-SEHOP) functions as a central hospital-based registry that systematically records all childhood cancer cases (<15 years) managed at paediatric oncology and haematology units. One of the RETI-SEHOP’s chief goals is to evaluate progress in the care of childhood cancer patients by analysing survival patterns and their evolution over time.

This study had two main aims: firstly, to provide an up-to-date description of childhood cancer survival in Spain for the 1999–2022 period, both overall and for a broad range of diagnostic entities, and, secondly, to analyse time trends in survival as an indirect means of assessing progress in the management of these diseases. A secondary aim was to document any survival differences by age and sex in the recent cohort (2009–2018) for comparison with the existing literature. Our results indicate that the period of 1999–2022 was marked by general improvements in childhood cancer outcomes in Spain, and we discuss how these changes relate to the implementation of new protocols and other developments in clinical practice.

## 2. Materials and Methods

### 2.1. Case Registration

The RETI-SEHOP is a central hospital-based cancer registry that coordinates a nationwide network of all paediatric oncology and haematology (SEHOP) units in Spain (44 centres as of 2025) [3,18]. New cases are actively reported by these units to the RETI-SEHOP. Follow-up data up to 5 years from diagnosis are provided by each unit upon request from the registry at 3 and 5 years. All data received are checked and coded before registration. Any queries arising during these case-by-case reviews, mainly with regard to tumour morphology, primary site, date inconsistencies, or patients’ usual residence at the time of diagnosis, are referred to the reporting centres for clarification prior to registration.

All cases included in this study were coded according to the International Classification of Diseases for Oncology, 3rd Edition (ICD-O-3.1) [19], and tumours were classified into diagnostic groups, subgroups, and further divisions as per the latest version of the International Childhood Cancer Classification (ICCC-3-2017) [20]. Date of diagnosis/incidence was defined as the earliest available date of diagnosis, considering all of the information that the registry received from all of the notifying centres related to each tumour.

Additionally, quality checks of registered cases are routinely performed, including the use of ENCR Quality Check Software (QCS) for this purpose. Follow-up information is cross-checked with the Spanish National Death Index (INDEF) as a complementary source. Death certificates are not used as a source of new case registration.

### 2.2. Study Population

The study population comprised all childhood cancer cases managed by SEHOP units and registered in the RETI-SEHOP, provided that they (i) had been diagnosed between 1 January 1999 and 31 December 2021; (ii) were under 15 years of age at the time of diagnosis; (iii) had a tumour classified in any valid category of the ICCC-3-2017; and (iv) had their usual place of residence in Spain at the date of diagnosis, as defined by Regulation no. 763/2008 of the European Commission [21]. Cases of myelodysplastic syndrome and other myeloproliferative diseases (Id) and miscellaneous lymphoreticular neoplasms (IId) were excluded from all analyses, following the criteria of previous publications [22,23]. Follow-up information up to 5 years after diagnosis was available for the incidence period 1999–2018. Cases diagnosed during 2019–2021 were followed up until 31 December 2022.

### 2.3. Statistical Analysis

Overall survival (OS) at 1, 3, and 5 years after diagnosis was estimated using the Kaplan–Meier method. For each set of cases, OS refers to absolute survival from all causes of death. For childhood cancer cases, absolute survival is almost equivalent to tumour cause-specific survival, especially in HIC [4]. The cohort approach was applied to cases diagnosed during the years 1999–2018, whereas the period approach was used for 2019–2022. The period method is used when follow-up for a recent cohort is not yet complete, as it provides the most up-to-date observed survival estimates available for that calendar period [24]. This method evaluates the survival experience of patients during the defined period using data from those diagnosed both within the period itself and up to 5 years earlier. In the case of the period of 2019–2022, follow-up data from cases diagnosed between 2014 and 2021 were used. Cases diagnosed in 2022 did not yet have follow-up data available and were therefore excluded, although the year 2022 was kept as a year of follow-up [25].

OS estimates were obtained by incidence cohort, age, and sex. The 1999–2018 period was divided into four 5-year cohorts, while a single estimate was obtained for the 2019–2022 period. Survival by age (<1 year, 1–4, 5–9, and 10–14 years) and sex (female/male) was analysed for the recent 2009–2018 incidence cohort. For some tumours, the <1-year and 1–4-year groups were pooled into a single 0–4-year group when either of the former contained fewer than 15 cases. Survival differences between sexes and age groups were assessed using log-rank tests. Survival estimates based on fewer than 15 cases are not reported in the tables.

Age-standardised survival estimates (<15 years) were calculated to account for demographic changes across the study period, allowing for more robust comparisons over time. For each tumour, age-specific weights were obtained from the case pool from the incidence period 1999–2021 (Table S1). Generally, age standardisation considered three age groups: 0–4, 5–9, and 10–14 years. However, adjacent age groups were combined (0–9 years or 5–14 years) when any group in a cohort contained fewer than 15 cases [26]. The 0–9- and 10–14-year groups were applied to tumours typically diagnosed at older ages, such as HL, bone sarcomas, intracranial/intraspinal and gonadal GCT, and epithelial neoplasms. Conversely, standardisation based on 0–4- and 5–14-year groups was used for ependymomas, medulloblastomas, neuroblastomas, and nephroblastomas. Due either to their scarcity or their incidence being concentrated within narrow age ranges, or both, age standardisation was not performed for retinoblastomas, hepatoblastomas, extracranial and extragonadal germ cell tumours (GCT), and thyroid carcinomas.

For a subset of clinically relevant tumours, survival trends were analysed with Cox proportional hazards models modified to accommodate joinpoints [27]. All cases diagnosed from 1999 to 2021 were included, with follow-up right-truncated at 5 years after diagnosis. For each tumour type, the number and location of joinpoints were determined by minimising the Bayesian information criterion (BIC). All models included the year of incidence as the explanatory variable, and, where possible, stratification by age groups was introduced to allow for computation of model-adjusted age-standardised survival estimates. Adjusted survival estimates were first calculated for each age group, and the overall age-standardised estimate and its standard error were subsequently derived using the corresponding weights (Table S1).

The absolute change in survival (AACS) represents the average yearly change in survival over a defined period, expressed in percentage points. AACS was used to quantify the magnitude of trends in the time series of adjusted 5-year survival estimates. Statistical significance of AACS was assessed through bootstrap resampling; for each tumour, the dataset was resampled 500 times, and the AACS was calculated for each bootstrap iteration using the selected model. Reported AACS values and their 95% confidence intervals (CI) correspond to the median and the 2.5th and 97.5th percentiles of the bootstrap distributions. Although cases diagnosed in 2022 were not included in the models, the adjusted survival time series were extended to 2022 by means of modelled prediction to match and compare them with the full study period.

All analyses were performed in R v4.4.1 (https://www.r-project.org/, accessed on 29 December 2025). Period survival estimates were obtained using the periodR package (v1.0-6) [28]. An ad hoc script was developed to fit age-stratified Cox models with up to two joinpoints to estimate age-standardised survival curves from individual case datasets (https://github.com/palf98/CoxJP, accessed on 29 December 2025).

## 3. Results

### 3.1. Brief Data Description

In the 1999–2021 diagnostic period, 20,534 cases with tumours classified under valid ICCC-3-2017 categories (Table S1) were registered in the RETI-SEHOP, with 8077 haematological neoplasms (39.3%) and 12,457 solid tumours (60.7%). Among the 5074 recorded central nervous system (CNS) tumours (including CNS GCT), 1657 were non-malignant (32.7%), though this proportion increased slightly across the study period, going from 31.7% in 1999–2010 to 33.4% in 2011–2021. This increase reached statistical significance (*p* = 0.037) in a linear model that tested the effect of diagnosis year on the proportion of non-malignant CNS cases. Second tumours accounted for 123 (0.6%) of all cases, and only two third tumours were registered during the 1999–2021 period for children aged 0–14 years. Our data complied with the IARC quality indicators for childhood cancer registries [29] (Table 1). Quality indicators by major diagnostic group are presented in Table S2.

### 3.2. Observed Survival

Follow-up at 5 years ranged from 96% to 98% for all tumours across the entire study period. In the 2019–2022 follow-up period, fewer than 3% of contributing cases were lost to follow-up before 5 years or the period’s closure date.

**Figure 1:**
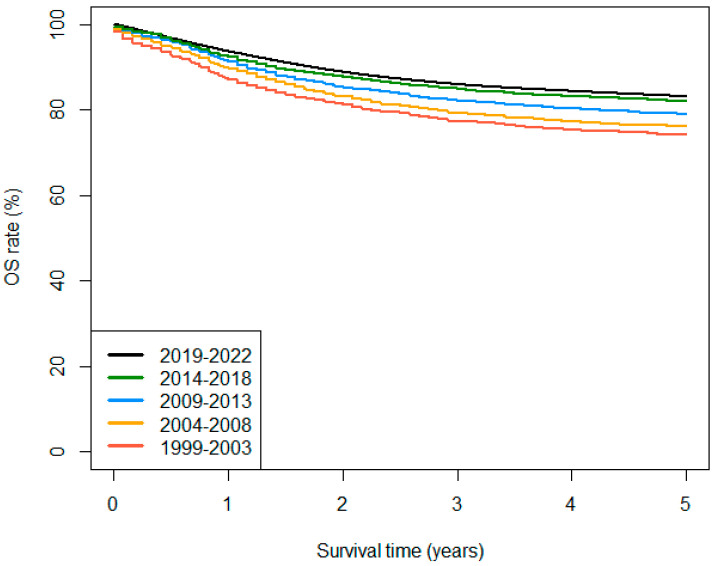
Observed survival curves for all tumours with malignant behaviour by cohort of diagnosis, 0–14 years. Period survival estimates are provided for the 2019–2022 cohort.

**Figure 2:**
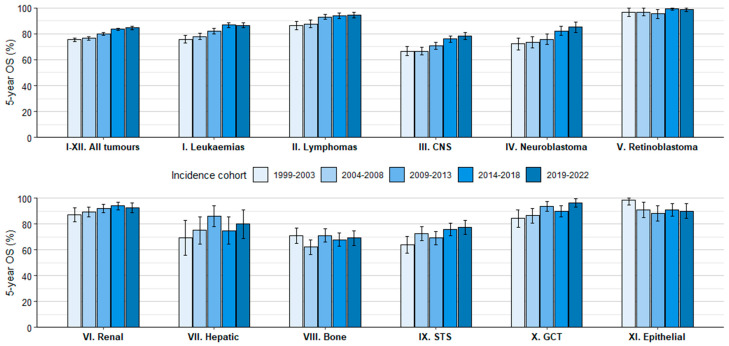
Observed 5-year OS rates by major diagnostic group and cohort of diagnosis, 0–14 years. Period survival estimates are provided for the 2019–2022 cohort.

During the study period, OS at 5 years increased by 9 percentage points for all tumours (ICCC-3-2017 groups I-XII) and all malignant tumours, reaching 84.6% and 83.1% in 2019–2022, respectively (Table 2, Figure 1). In 2019–2022, 5-year OS appears to have consolidated above 90% for Hodgkin lymphomas (HL), Burkitt lymphomas (BL), retinoblastomas, renal tumours, and GCT. In contrast, survival remained comparatively low for intracranial and intraspinal embryonal tumours, other gliomas (a category formed by non-ependymoma, non-astrocytoma gliomas), and malignant bone tumours. Figure 2 summarises observed 5-year OS rates by main diagnostic group and cohort of diagnosis. A more detailed overview of tabulated survival results, including the number of cases, follow-up percentages at 5 years, and OS at 1 and 3 years by diagnostic group, is shown in Tables S3–S7.

Remarkable survival improvements were observed for cases of haematological malignancies between 1999–2003 and 2019–2022. Comparing the first and the last estimates of the series, 5-year risk of death decreased by 45% for leukaemias and 59% for lymphomas (Table 2). For BL specifically, 5-year risk of death declined by approximately 80%. Conversely, progress in acute myeloid leukaemia (AML) has been comparatively modest within haematological neoplasms, leaving substantial room for further improvement.

For solid tumours, the picture is more heterogeneous. At one end of the spectrum, 5-year risk of death fell close to zero in astrocytomas (non-malignant), retinoblastomas, and gonadal GCT, all of which already had high survival rates at the beginning of the study period. At the other end, little or no improvement was observed for other gliomas and malignant bone tumours, which started with some of the lowest survival rates among the tumours studied.

For all cancers combined, OS up to 5 years differed significantly across age groups (Table 3). In general, observed survival was highest for children aged 1–4 years and lowest among those aged 10–14 years. By specific diagnostics, significant differences by age group were identified for lymphoid leukaemias (LL), CNS tumours, neuroblastomas, hepatoblastomas, and epithelial neoplasms. For CNS tumours, differences disappeared when analysing non-malignant cases. The number of cases by age group used to obtain the calculations in Table 3 is shown in Table S8.

In general, survival rates were homogeneous between males and females (Table S9). Among the 38 tumour categories studied, a statistically significant difference in survival by sex was observed solely for non-Hodgkin lymphomas (NHL) (except for Burkitt). Males, who accounted for almost 70% of these cases, had a 5-year OS rate of 90.5% in the 2009–2018 cohort, compared to 81.9% in females

### 3.3. Survival Trends

Age-standardised survival rates are shown by cohort and tumour type in Table S10, including the 2019–2022 follow-up period. Because case population weights are used for the standardisations, estimates can be interpreted on the scale of observed 5-year survival for all ages (0–14 years).

**Figure 3:**
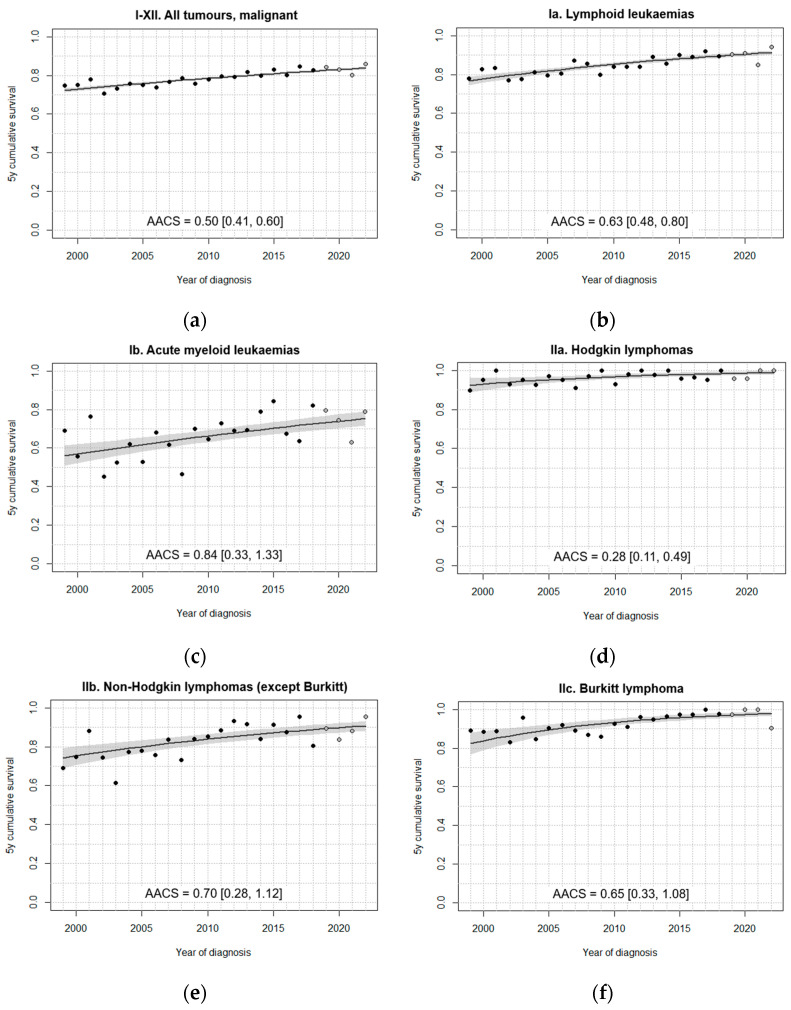
Fitted trends in age-standardised 5-year survival for (**a**) all tumours and (**b**–**f**) haematological malignancies diagnosed in Spain, 1999–2022, 0–14 years. Black dots correspond to annual cohort estimates, while grey dots correspond to annual period estimates. Shaded areas represent 95% confidence bands around the fitted curves. AACS [95% CI]: average absolute change in survival.

**Figure 4:**
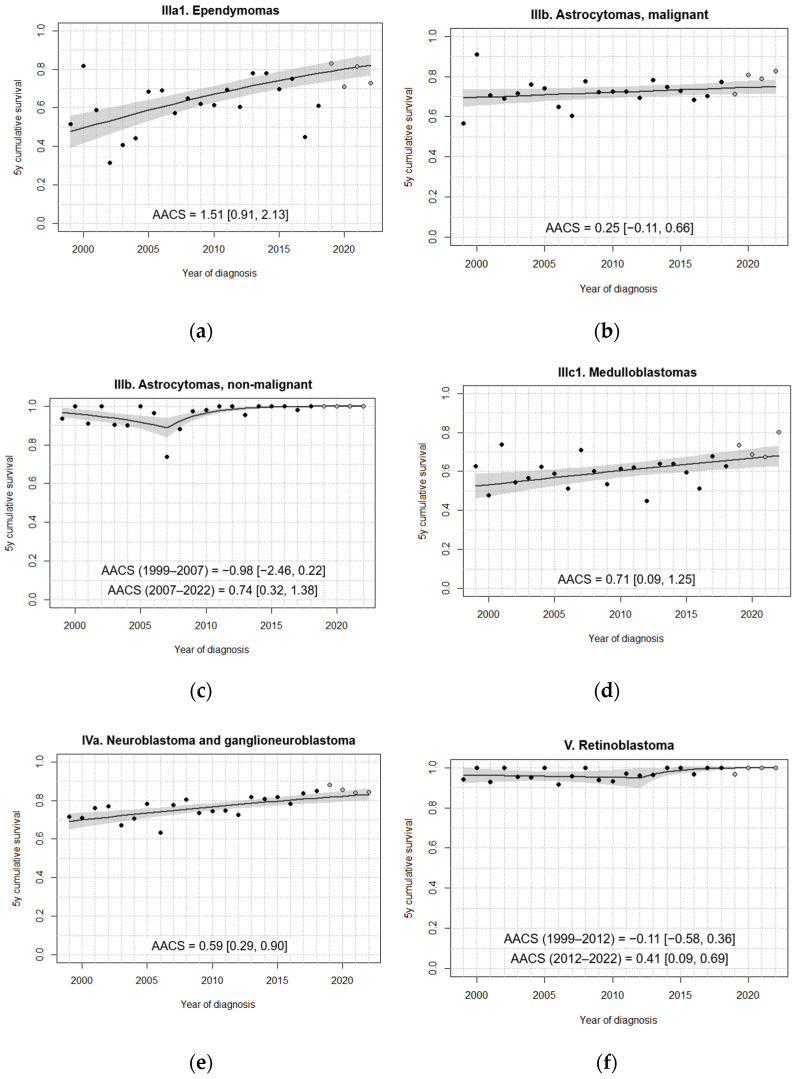
Fitted trends in 5-year survival for (**a**–**d**) selected brain tumours, (**e**) neuroblastomas, and (**f**) retinoblastomas diagnosed in Spain, 1999–2022, 0–14 years. Age standardisation was performed in all cases except for (**f**) retinoblastomas. Black dots correspond to annual cohort estimates, while grey dots correspond to annual period estimates. Shaded areas represent 95% confidence bands around the fitted curves. AACS [95% CI]: average absolute change in survival.

**Figure 5:**
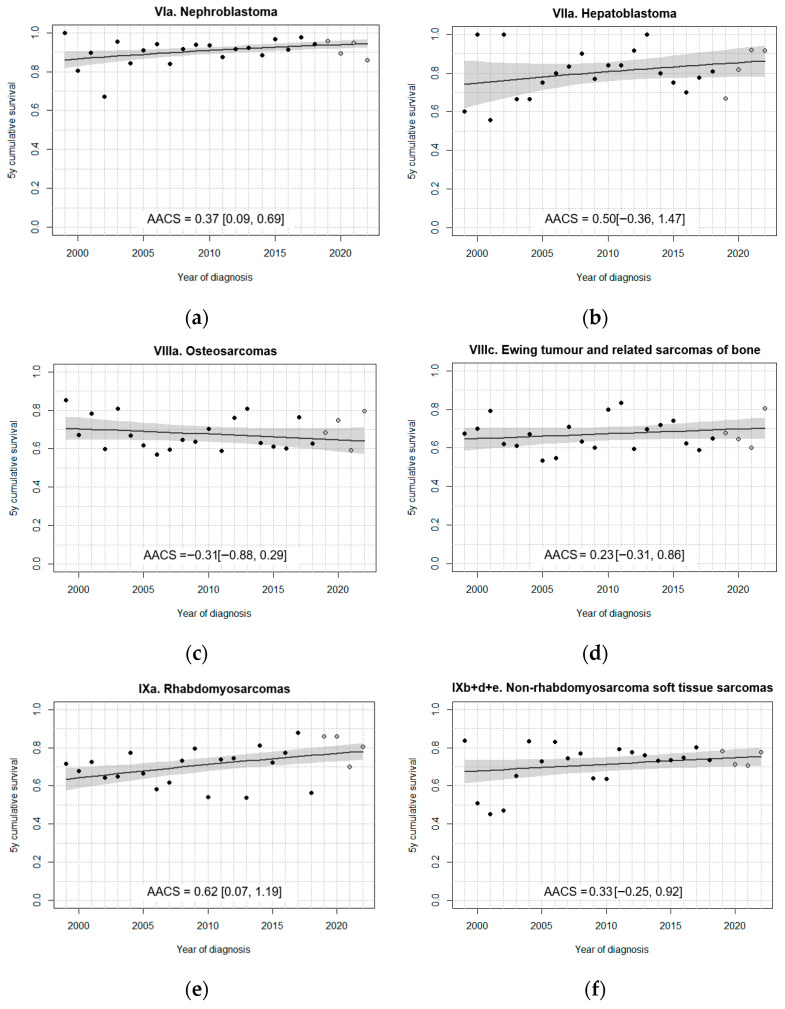
Fitted trends in 5-year survival for (**a**) nephroblastoma, (**b**) hepatoblastoma, (**c**,**d**) bone tumours, and (**e**,**f**) soft tissue sarcomas diagnosed in Spain, 1999–2022, 0–14 years. Age standardisation was performed in all cases except for (**b**) hepatoblastomas. Black dots correspond to annual cohort estimates, while grey dots correspond to annual period estimates. Shaded areas represent 95% confidence bands around the fitted curves. AACS [95% CI]: average absolute change in survival.

**Figure 6:**
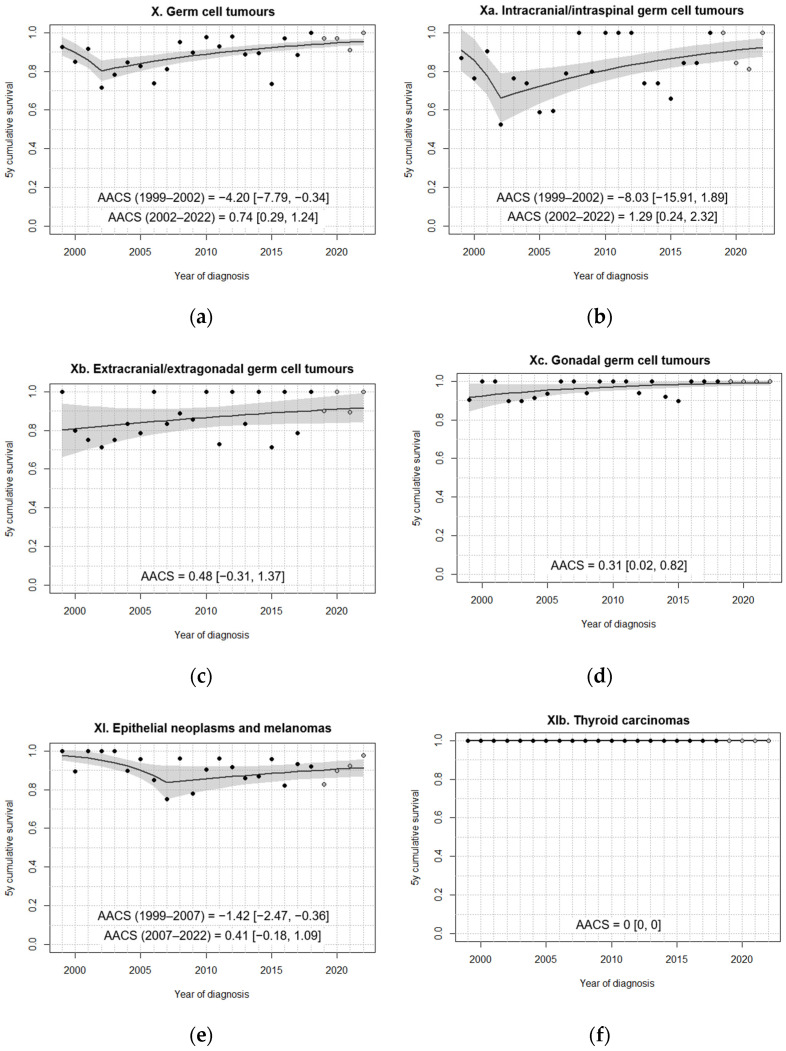
Fitted trends in 5-year survival for (**a**–**d**) germ cell tumours and (**e**,**f**) epithelial neoplasms diagnosed in Spain, 1999–2022, 0–14 years. Age standardisation was not performed for (**c**) extracranial/extragonadal germ cell tumours and (**f**) thyroid carcinomas. Black dots correspond to annual cohort estimates, while grey dots correspond to annual period estimates. Shaded areas represent 95% confidence bands around the fitted curves. AACS [95% CI]: average absolute change in survival.

In general, fitted models did not identify change points in the 5-year survival trend over the study period (Figure 3, Figure 4, Figure 5 and Figure 6). A single change point was only identified for non-malignant astrocytomas, retinoblastomas (Figure 4), GCT and intracranial/intraspinal GCT in particular, and epithelial tumours (Figure 6), but in no case did the models that minimised BIC identify more than one joinpoint.

For all tumours with malignant behaviour combined, the fitted model identified a significant increase in age-standardised survival between 1999 and 2022 (Figure 3a), achieving an increase of approximately one percentage point every two years on average. Given the large number of cases included and the nearly linear progression of the observed annual hazard estimates, the model seems to correctly capture survival variation throughout the period.

All analysed subgroups of haematological malignancies experienced statistically significant upward trends across the study period (Figure 3b–f). Fitted 5-year survival estimates for LL (Figure 3b) and non-Burkitt NHL (Figure 3e) surpassed 90% in about 2019. AML experienced the highest AACS among haematological neoplasms over the study period, but their survival still lags well behind that of the other subgroups (Figure 3c). Age-standardised 5-year survival in BL increased to almost 100% by 2022, with an estimated AACS of 0.65 over the series of 24 years, which is exceptionally high given that fitted survival was already around 84% at the start of the series (Figure 3f).

In contrast to the relatively homogeneous trends seen in haematological malignancies, survival patterns among solid tumours, including brain tumours, were more diverse (Figure 4, Figure 5 and Figure 6). Among all tumours studied, ependymomas registered the lowest fitted 5-year survival rates at the beginning of the study period (Figure 4a). However, they experienced the steepest absolute increase in 5-year survival by more than one percentage point every year. Throughout the period, approximately 7% of ependymomas were non-malignant, with this proportion remaining relatively stable. For malignant astrocytomas, no statistically significant improvement in survival could be established, as the 95% CI of the AACS included 0 (Figure 4b). In contrast, non-malignant astrocytomas showed significant improvement from 2007 onwards, with fitted 5-year survival rates remaining nearly at 100% after 2015, consistent with the observed values (Figure 4c). For medulloblastomas, observed annual estimates showed substantial variability, but the model was able to identify a significantly positive AACS, estimated at around 0.71 (Figure 4d).

Overall, extracranial embryonal tumours displayed positive trends. Both fitted and observed data show a consistent increase in survival for neuroblastoma cases throughout the period (Figure 4e). Like non-malignant astrocytomas, retinoblastomas registered a phase of significant survival improvement beginning after 2012, following an earlier period of stagnation (Figure 4f). For nephroblastomas, the fitted model identified a modest, albeit significant, upward trend in 5-year survival, with survival exceeding 90% after 2008 (Figure 5a). Unlike the rest of the analysed embryonal tumours, no significant improvements in survival were found for hepatoblastomas, possibly due to the number of cases being insufficient to detect a slight increase (Figure 5b).

Among the analysed sarcomas, only rhabdomyosarcomas (RMS) exhibited a significant increase in fitted age-standardised 5-year survival (Figure 5e), as opposed to osteosarcomas (Figure 5c), Ewing sarcomas of bone (Figure 5d), and non-RMS soft tissue sarcomas (STS) (Figure 5f). The fitted model for RMS indicated an annual increase of 0.62 percentage points in age-standardised 5-year survival, though the proximity of the lower end of the 95% CI to 0 suggests that this improvement should be interpreted with caution. In the case of osteosarcomas, though not statistically significant, the model fitted a slightly negative survival trend between 1999 and 2022.

For all GCTs combined, the model identified a significant decline in age-standardised 5-year survival during the early years of the study period (1999–2002), followed by a positive trend that eventually recovered and surpassed the initial loss in survival (Figure 6a). This change point was located near the beginning of the series, leaving only three years prior to it, which was the minimum allowed. The negative trend in these early years appeared to be mostly influenced by extragonadal GCT (Figure 6b,c). In the case of intracranial/intraspinal GCT, the same change point was identified, with a very steep decrease in survival between 1999 and 2002 (Figure 6b). However, this negative trend was not statistically significant due to the low number of cases included in this subgroup. For extracranial/extragonadal GCT, no statistically significant trends were identified (Figure 6c). A mild improvement was observed in gonadal GCT, with 5-year survival slowly approaching 100% by the end of the period (Figure 6d).

For epithelial neoplasms, a significant decrease in survival was identified between 1999 and 2007, followed by a period of non-significant improvement (Figure 6e). Notably, the number of registered epithelial tumour cases, i.e., the number of patients attending SEHOP units, rose substantially over the study period (Table S7). This broader inclusion of cases, potentially involving patients with different prognostic profiles, may partly explain the initial decline in survival. Because no deaths were observed for thyroid carcinomas during the study period, no trends could be analysed (Figure 6f).

## 4. Discussion

This study analyses survival outcomes and time trends based on more than 20,000 children (0–14 years) treated at SEHOP units, which provides nationwide coverage. The 24-year study period offers a sufficiently long time frame to combine historical and recent data, allowing for analysis of long-term survival trends. We used the period approach to estimate 5-year survival rates up to 2022, with the aim of providing the most up-to-date observed survival results possible [30].

On average, approximately 3% of cases were lost to follow-up at 5 years. This finding was applicable to most diagnostic groups, except for epithelial tumours, for which the proportion of cases lost to follow-up ranged from 10 to 23%. For these tumours in particular, survival results should be interpreted with caution, as censoring might have been associated with prognostic differences. Among the main reasons for loss to follow-up is that notifying units may lose contact with patients after cure or transfer to non-paediatric units.

In Spain, the vast majority of children with cancer are treated at SEHOP units, especially in recent years. These units handled roughly 80% of incident cases during 1999–2009 and more than 90% after 2009 [3]. A proportion of childhood cancer cases continues to be managed in non-paediatric units, such as general surgery, haematology, and dermatology departments; this is especially true of haematological neoplasms and epithelial tumours. In the Spanish context, collaborative efforts between regional population-based cancer registries and the RETI-SEHOP are being made to achieve the most representative datasets possible for such areas through record linkage.

Among SEHOP units, 5-year OS for all tumours exceeded 80% in the 2009–2013 cohort and neared 85% in the period of 2019–2022 for children aged 0–14 years. These figures fit comparatively well with published results from other HICs. In EUROCARE-6, 5-year survival in 2010–2014 for the pool of all participating European registries was estimated at 81.3% (period approach) [4]. The nationwide Italian hospital-based childhood cancer registry reported 5-year survival of up to 84% in 2009–2017 (period approach) [7]. In the Netherlands, childhood cancer survival stood at 83.7% in 2010–2015 and at 81.1% for tumours of malignant behaviour only [31]. In France, 5-year survival increased from 80.8% in 2004–2008 to 84.6% in 2010–2016 [32]. England’s National Health Service (NHS) published a series of annual survival estimates for childhood cancer in 2002–2020, surpassing the 80% mark after 2006 and the 85% mark after 2016 (period approach: 2017–2020) [33]. It should be noted that the most up-to-date survival estimates provided by cancer registries are often released on their institutional websites well in advance of publications from international collaborative projects, such as EUROCARE. However, this mode of dissemination may limit long-term accessibility if such results are not published in public repositories or peer-reviewed journals.

In our 2009–2018 cohort, survival was broadly similar between males and females, with a significant difference observed only for NHL (except for Burkitt), where males registered higher 5-year survival than did females (90.5% vs. 81.9%). Other studies have reported sex-related differences in several tumour types [34], but most of these differences disappeared when adjusting for age, histology, site, and stage, except in the case of ependymomas [35]. Overall, sex appears to have a limited influence on childhood cancer survival. Any observed differences are likely small and may diminish over time as therapeutic approaches improve for disease subtypes that differ in prevalence between the sexes.

In contrast, age at diagnosis showed a clear association with survival, which was especially stark for some tumours. Three distinct age-related survival patterns were observed: (i) survival peaking at 1–4 years (LL, malignant astrocytomas, hepatic tumours); (ii) survival increasing with age (ependymomas and choroid plexus tumour, CNS embryonal tumours, epithelial tumours); and (iii) survival decreasing with age (other gliomas, neuroblastomas). These results are in line with the findings for the French 2000–2008 cohort [34].

Among LL, age disparities have persisted over time due to a lack of progress in infant disease. In children under 1 year of age, LL presents unique biological and clinical challenges, such as *KMT2A* rearrangement mutations and increased treatment toxicity [36,37]. Furthermore, survival decreased slightly for children over 9 years old, replicating the pattern observed in CONCORD-2 [38]. For malignant astrocytomas, glioblastoma represented the morphology with the poorest outcomes in our cohort, comprising <10% of cases among children aged 1–4 years but around 20% in other groups. Conversely, malignant gliomas of the optic nerve, a histology with excellent survival, were the most prevalent among cases 1–4 years old (>40%).

Complete resection for ependymomas below 3 years of age is known to be challenging, which is partly responsible for the poorer outcomes in this age bracket. Treatment regimens for intracranial ependymoma in this age group are characterised by avoidance or delay of radiotherapy to reduce the risk of cognitive sequelae and secondary tumours [39,40]. For neuroblastomas, older age at diagnosis correlates with advanced disease, but survival differences persist even after adjustment for stage [41]. On the whole, survival variations by age probably reflect a combination of biological heterogeneity, treatment tolerance, and surgical feasibility. These findings highlight the need for continued biologically based stratification in paediatric oncology research.

Overall, fitted trends in age-standardised survival between 1999 and 2022 were predominantly linear for Spain, with no change points for the most part. In a study analysing paediatric cancer survival trends with SEER data by main diagnostic group, no joinpoints were identified after 1999 [42], which suggests that progress over recent decades has generally advanced at a steady pace.

Remarkable progress has been made in paediatric haematological neoplasms during the study period. Observed trends closely resembled those published recently for a selection of Spanish regions in a population-based study (2000–2016) [43]. This similarity supports the contention that to a greater or lesser degree, their results, covering almost 65% of the Spanish paediatric population, can be extrapolated to the national level.

Survival trends for LL indicated steady improvement across the study period, which was characterised by a succession of two branches of national protocols that were unified in 2013: SHOP/ALL-99 (1999–2005), SHOP/ALL-05 (2005–2013), PETHEMA ALL-IR 96 (1996–2013), PETHEMA ALL-LR 2001 (2001–2013), and PETHEMA ALL-HR 2005 (2005–2013). In 2013, LAL-SEHOP-PETHEMA-2013 unified national protocols, thereby integrating the experience of the SHOP and PETHEMA groups. These protocols have achieved a reduction in deaths and relapses in all risk strata of non-infant LL. In the case of infants, even though Spain did not participate in the European INTERFANT-06 trial (EudraCT 2005-004599-19), most units nevertheless adopted its scheme from 2007 onwards. However, results of the INTERFANT-06 phase III trial showed no significant improvements with respect to the previous INTERFANT-99 protocol [44]. Recently, the incorporation of blinatumomab into the INTERFANT-06 backbone showed potential to enhance outcomes for infants with ALL [45]. Despite overall progress in LL management in Spain, 5-year survival has yet to surpass 90%, whereas other European countries reached this threshold years ago [4,6,46]. This gap prompted SEHOP to prioritise first-line treatment in prospective clinical trials conducted by European cooperative study groups, rather than relying solely on guideline-based therapeutic protocols.

In the case of AML, treatment regimens during the study period specialised in Down syndrome (DS) cases, acute promyelocytic leukaemias (APL), and non-DS AML cases. DS cases were treated following their BFM-98 protocol branch until 2007. After that, the strategy of the ML-DS-2006 trial (EudraCT 2007-006219-2) was adopted. However, the ML-DS-2006 trial did not report an increase in survival for DS cases, which was already high [47]. For APL, specific national protocols were used throughout the study period (PETHEMA-LPA-96/2005/2012/2017), optimising risk-adapted strategies based on the use of ATRA [48]. PETHEMA-LPA-2017 sought to improve outcomes by introducing ATO + ATRA therapy for low- and intermediate-risk cases. In 2017, Spanish centres began participating in the NOPHO-DBH-AML-2012 phase III trial (EudraCT 2012-002934-35) for non-DS AML cases [49], though some units had already started to adhere to their protocol several years earlier. In all likelihood, improved risk stratification, novel therapies, and advances in supportive care and haematopoietic stem cell transplantation contributed to rising cure rates, consolidating 5-year OS above 70%, in line with other European countries. [6,7,50]. Looking ahead, further improvements for AML will depend on the cooperation of international collaborative groups, enabling the design of large prospective trials, the integration of biology-driven risk stratification, and the incorporation of targeted therapeutic approaches [51].

The 1999–2022 period proved to be very positive for lymphomas, with only NHL remaining below 90% 5-year OS. HL showed excellent survival from the start of the period, yet some improvements were still observed, especially around 2008, as shown by the OS estimates by cohort. Around this time, Spanish centres shifted from national protocols to EuroNET-PHL-C1 (EudraCT 2006-000995-33), which omitted radiotherapy for early-stage cases with good response to chemotherapy, ultimately reducing toxicity [52]. This change might partly explain the observed increase in survival. Spain has actively participated in both EuroNET-PHL-C1 and EuroNET-PHL-C2 (EudraCT 2012-004053-88), the latter covering the most recent years of our cohort since 2016.

Given the marked biological and therapeutic heterogeneity of NHL, trends observed likely reflect a composite of different changes in clinical practice. Major NHL subtypes comprise mature B-cell lymphoma, lymphoblastic lymphoma (LBL), and anaplastic large cell lymphoma (ALCL). In the late 1990s, a major milestone in our country was joining the European Inter-Group for Childhood Non-Hodgkin Lymphoma (EICNHL), which allowed active participation in international protocols and cooperative clinical trials. These trials incorporated new biomarkers, risk stratification strategies, and novel therapies, all contributing to improved survival outcomes.

Regarding B-mature NHL, including BL, Spain adopted the LMB-89 [53] dose-dense treatment strategy in the early 2000s, achieving better outcomes than earlier national protocols. Subsequent participation in the phase III INTER-NHL-B-2010 trial (NCT01516580) demonstrated that adding rituximab to LMB chemotherapy significantly improved survival in high-risk patients, becoming the standard of care in many HICs [54]. Although Spain did not participate in Euro-LB-02 (NCT00275106) [55], its control arm was adopted as the national standard for LBL, avoiding CNS irradiation but yielding lower event-free survival than earlier BFM studies [56]. Since 2020, Spain has participated in the LBL2018 trial (NCT04043494), which incorporates molecular risk stratification (*NOTCH1*/*FBXW7*). For ALCL, Spanish centres did not participate in the ALCL99 trial (NCT00006455) but adopted its protocol as the national standard of care in the early 2000s, as it represented the option with the lowest treatment intensity among the available regimens. Given the strong prognostic value of *NPM1-ALK*-based biomarkers in ALCL [57], Spain has recently incorporated centralised assessment into routine practice to refine risk stratification and guide treatment intensity, particularly in the context of emerging targeted therapies.

Despite excellent survival for NHL overall, outcomes in relapsed/refractory (r/r) cases remain poor [58]. Only one clinical trial within the eicnhl addressed r/r NHL from 1999 to 2021, the ALCL Relapse trial (NCT00317408) [59], whose recommendations were followed by Spanish centres until targeted therapies became available after 2022. For r/r B-NHL, rituximab combined with ICE followed by autologous SCT has been the standard of care, with limited success in some entities [60]. At the time of writing this manuscript, the Glo-B-NHL study (EudraCT 2021-004283-10), a prospective, risk-adapted, cooperative clinical trial focusing on r/r B-NHL, is open for recruitment [61].

We performed separate analyses for malignant and non-malignant CNS tumours to avoid bias, as the proportion of registered non-malignant cases increased during the study period. A previous study covering the period of 1991–2005 reported that survival for childhood CNS tumours in Spain and its major subtypes was stagnant and below that of Europe [62]. In our study, we observed positive trends in the 1999–2022 period for ependymomas, non-malignant astrocytomas, and medulloblastomas, narrowing the gap with Europe. The 5-year OS for malignant CNS tumours reached 63.7% in 2014–2018 and is expected to reach 66.2% or more in the 2019–2022 cohort, which compares relatively well with the 66% reported for Germany in 2011–2016 [6].

Between 1999 and 2005, CNS cases under 3 years of age were treated with a single national protocol. At the same time, ependymomas over 3 years were managed following the SIOP Ependymoma I protocol, which emphasised total resection and validated the effectiveness of VEC chemotherapy in residual tumours [63]. In parallel, it became increasingly clear that post-operative radiotherapy in cases under 3 years of age enhanced disease control [64]. From 2015 onwards, most ependymomas regardless of age followed the SIOP Ependymoma II protocol (EudraCT 2013-002766-39), which was widely adopted in Spanish centres. This protocol avoided radiotherapy in children below 1 year of age, planning for delays and temporary control with chemotherapy to prevent cognitive sequelae. Further improvements can be expected after 2022, driven by the COG ACNS0121 phase III (NCT00027846) and SIOP Epend II phase II/III trials. In addition, new European guidelines now stratify treatment using specific molecular markers [40].

Our study identified a period of stable survival for non-malignant astrocytomas from 1999 to 2007, followed by a rising trend up to 2022. The joinpoint occurs in a year with an abnormally low 5-year survival, which may have exaggerated the AACS in the second segment. Nonetheless, an improvement is evident in the second half of the period, coinciding with more exhaustive capture of non-malignant CNS cases. Non-malignant astrocytomas were treated with the SIOP-LGG-1995 and SIOP-LGG-2004 protocols. The SIOP-LGG-2004 trial (EudraCT 2005-005377-29) recruited patients, including Spanish cases, to test the addition of etoposide to classic vincristine + carboplatin induction [65]. We suggest that this new protocol, together with surgical refinements, contributed to the increase in survival, which approached 100% after 2010. Conversely, we report no significant progress in outcomes for malignant astrocytomas. German HIT-GBM-D and HIT-HGG-2007 (EudraCT 2007-000128-42) protocols were sporadically used in Spanish units. From 2011 to 2016, the HERBY trial (EudraCT 2010-022189-28) recruited high-grade gliomas to test the addition of bevacizumab to radiotherapy + temozolomide treatment but was halted due to high toxicity, with no added benefit [66]. High-grade gliomas continue to lag behind in survival progress, and broader collaborative efforts may be needed to advance in this field.

Medulloblastomas displayed the second most marked survival improvement among brain tumours in terms of AACS. PNET-SIOP-3, PNET-SIOP-4, and PNET-SIOP-5 (EudraCT 2011-004868-30) were the most frequent protocols in our cohort. Since 2009, high-risk cases under 4 years have generally been treated following the HEAD-START-I, II, and III protocols, which have likely contributed to improve outcomes and reduce late effects using brief and intensive chemotherapy [67,68]. Since 2017, a non-negligible proportion of high-risk cases over 3 years has been treated following COG ACNS-0332/0334 strategies (NCT00392327/NCT00336024), which similarly achieved better outcomes by combining radiotherapy and carboplatin treatment [69]. Nevertheless, survival remains limited, and long-term quality of life has yet to be improved.

There was significant progress in outcomes for all embryonal tumours, except for hepatoblastomas. Turning to neuroblastoma, a recent Spanish study (2000–2017) suggested positive effects of the HR-NBL-1 (EudraCT 2006-001489-17) and LINES (NCT01728155) trials and highlighted that improvements were mostly concentrated among locoregional cases [70]. Our data indicate that progress continued through 2022, reaching a 5-year OS of 85%. For retinoblastomas, survival consistently approached 100% after the joinpoint in 2012. In Spain, retinoblastomas were managed by five highly specialised ophthalmology services throughout the study period. Key developments around 2012 included the use of intraarterial chemotherapy as first-line treatment and intravitreous/intracameral chemotherapy to eliminate residual disease, enabling a shift from systemic to eye-directed chemotherapies. Paired with the avoidance of enucleation, these techniques improved both survival and vision-preservation rates [71]. In the case of nephroblastomas, steady improvement occurred from 1999 to 2022, a period with strong adherence of SEHOP units to SIOP protocols (SIOP-93, SIOP-01, and UMBRELLA SIOP-RTSG 2016). SIOP-93/01 trials highlighted the importance of preoperative chemotherapy and proper postoperative risk stratification in the management of non-metastatic cases [72]. Nevertheless, a small subset of high-risk nephroblastomas remained refractory. The UMBRELLA protocol (EudraCT 2016-004180-39) unifies the management of nephroblastomas and other renal tumours by performing centralised diagnostic evaluation, which determines risk stratification and directs the recommended treatment [73]. With respect to hepatoblastomas, Spanish cases were treated by successively following the European SIOPEL-3, -4, and -6 protocols. While SIOPEL-3 and -4 introduced significant progress in outcomes for high-risk patients by intensifying preoperative chemotherapy with cisplatin [74,75], SIOPEL-6 (EudraCT 2007-002402-21) focused on reducing cisplatin-induced ototoxicity in standard-risk patients with sodium thiosulfate [76]. The PHITT trial (NCT03017326) protocol, adopted in Spain around 2018, employs the CHIC risk stratification system based on PRETEXT staging [77,78,79], but its implementation is too recent to evaluate with our cohort. Due to their rarity, the positive trend modelled for hepatoblastomas in our study has a wide CI, but we suggest that this positive trend may consolidate in the coming years.

As in other recent cancer registry studies, we report no significant progress for bone sarcomas [6,7,31,42]. During the period of 1999–2022, Spanish osteosarcoma cases were treated according to national protocols (SEOP-SO-95/2001/2010). Spain did not participate in the EURAMOS-1 trial (NCT00134030), which ultimately did not significantly improve outcomes [80]. Since 2014, non-metastatic extremity cases have been enrolled in the GEIS-33 national trial (NCT04383288), which sought to identify molecular markers for biological stratification [81]. The use of mifamurtide in adjuvant chemotherapy has recently shown promising results for localised ABCB1/P-glycoprotein-positive cases [82]. Yet despite the availability of this drug in Spain since 2010, we found no significant impact on population-level outcomes. With regard to Ewing sarcomas, national protocols predominated until the introduction of EuroEwing-2012 (EudraCT 2012-002107-17). Most cases in the 1999–2015 period were treated according to Ewing-SEOP-95/99/01. EuroEwing-2012 became predominant after 2016, with some overlap with the national GEIS-21 trial (NCT01696669). GEIS-21 explored the use of gemcitabine + docetaxel cycles in high-risk patients [83]. EuroEwing-2012 subsequently introduced VDC + IE, which demonstrated survival advantages in children under 14 years [84]. Even so, our cohort reflects no clear progress between 1999 and 2022.

RMSs and other STSs were managed under SIOPE protocols throughout the study period. RMSs were treated according to the MMT-95 protocol until 2005 and under the EpSSG-RMS-2005 protocol (EudraCT 2005-000217-35) from 2005 to 2021. The FaR-RMS protocol (EudraCT 2018-000515-24) was first used in 2021. These successive cooperative trials progressively refined risk-adapted multimodal treatment, reducing the chemotherapy burden where possible [85,86]. Survival of RMS cases in Spain increased significantly over time and is on par with other European countries [4]. Non-metastatic non-RMS STS, except for Ewing and Askin tumours, were treated largely under the EpSSG-NRSTS-2005 protocol (EudraCT 2005-001139-31) for most of the study period. This protocol unified treatment across Europe and improved risk stratification. Progress remains limited, however, partly due to the heterogeneity and poor chemosensitivity of these tumours [87]. Regarding metastatic cases, no major breakthroughs were introduced during the study period [88]. The positive but not significant trend in our findings highlights the need for further advancements in non-RMS STS management, including subtype-focused research and careful adaptation of adult trial findings to paediatric patients.

Overall, GCT displayed favourable trends. Children with CNS GCT in Spain were generally managed according to SIOP-CNS-GCT-96 until 2010 and thereafter with SIOP-CNS-GCT-II (EudraCT 2009-018072-33). Throughout the study period, craniospinal irradiation remained the standard for germinomas, while non-germinomatous GCT require multimodal therapy, as demonstrated by the SIOP-CNS-96 trial [89,90]. The SIOP-CNS-GCT-II trial sought to adjust the radiotherapy dose for germinomas to maintain already excellent outcomes and to improve survival in high-risk non-germinomatous GCT through chemotherapy dose escalation, which may partly explain the positive trend observed after 2007. Survival for gonadal GCT approached 100%, with no deaths registered for cases diagnosed after 2015. Although we observed no significant changes in survival for extracranial/extragonadal GCT cases, it is difficult to ascertain the accuracy of this, given that this subgroup had fewer than 10 cases annually. Moreover, not performing age standardisation for this rare subgroup may have led to an underestimation of the trend, as infants not only show higher survival but also became a lower proportion of all children after 2010.

In the case of epithelial tumours, the identified negative trend in 1999–2007 most likely reflects an artifact of insufficient follow-up completeness and the increase in coverage by paediatric oncologists, which probably affected the composition of diagnostics analysed over time (Table S11). Currently, epithelial tumours make up the main ICCC-3 group with lowest registration completeness in the RETI-SEHOP [3], suggesting that many are still being managed by other services, especially thyroid carcinomas and localised malignant melanomas.

In recent decades, improving quality of life has become a major priority for clinicians, especially in the case of tumours for which survival is already high or excellent. There is also a need to evaluate survivorship in the growing population of childhood cancer survivors [91]. However, most cancer registries currently lack the capacity to record such data systematically, given the multidimensional nature of quality of life, although long-term excess mortality might serve as a reasonable provisional proxy [92]. Our findings underscore the importance of incorporating such data into HIC cancer registries in order to keep monitoring progress, especially for tumours already approaching 100% long-term disease-related survival.

As for the strengths of our study, we analysed survival over a 24-year period for all children with cancer attended by paediatric oncologists and haematologists in Spain, providing up-to-date estimates through the period approach. We studied survival trends using Cox–joinpoint models, which yielded meaningful results overall. These models captured trends consistently across the entire period, as residuals by diagnosis year were relatively homogeneous. Additionally, we modelled age-standardised survival to minimise any bias introduced by demographic changes. Finally, despite changes in histopathological and ICD-O classifications over time, comparability across the 24-year period was ensured by systematic back-coding of all cases.

With respect to limitations, survival figures for 2019–2022 can be expected to be slightly underestimated, as has been shown to happen for period estimates in a context of continuous progress in care [30]. Joinpoints identify trend changes well but fail to identify abrupt additive changes, as can occur with therapeutic breakthroughs. Regarding registration, non-malignant CNS and epithelial tumour registration completeness increased across the study period, hindering longitudinal analysis. Though a primary benchmark, 5-year survival cannot suffice to describe long-term survival and is even less informative for assessing survivors’ quality of life. Additionally, we could not obtain event-free survival, a preferred metric for outcome evaluation in some diagnostics, because relapse information is not systematically recorded by the registry.

## 5. Conclusions

Childhood cancer 5-year survival in Spain is currently approaching 85%, in line with other European countries. Our retrospective analysis of the SEHOP network of paediatric oncology and haematology units across the period of 1999–2022 reveals substantial survival improvements for most childhood cancer diagnostics. Survival increases have been generally steady, reflecting a cumulative effect of advances in clinical practice. More precise diagnostics and better-tailored risk-adapted treatments are likely responsible for much of this progress. However, there are still several tumours with unfavourable outcomes that did not show significant improvements, such as malignant astrocytomas and bone sarcomas. Addressing tumours with persistently poor survival and reducing late effects remain key challenges for Spanish paediatric oncology in the coming years.

## Figures and Tables

**Table 1:** Compliance of RETI-SEHOP registration with IARC quality standards for childhood cancer registries, 1999–2021.

IARC Quality Indicators	Recommended Values	RETI-SEHOP, 1999–2021
Microscopically verified cases (MV%)	85–98	89.5
Cases ascertained from death certificate only (DCO%)	<5	-
Proportion of unspecified cases (NOS%) *	<10	2.1
Cases aged < 1-year (%)	5–15	10.9
Non-malignant CNS cases (%) †	20–40	32.7

* Percentage of cases classified as IIe, IIIf, VIc, VIIc, VIIIe, IXe, Xe, XIf, and XIIb and leukaemias with histology codes 8000, 8800, and 9800. † Comprises diagnostic group III and subgroup Xa.

**Table 2:** Observed 5-year survival and 95% confidence interval by diagnostic group, 0–14 years.

Diagnostic Group	5-Year OS Rate (%) [95% CI]				
	1999–2003	2004–2008	2009–2013	2014–2018	2019–2022 *
I-XII. All tumours	75.4 [73.9, 76.9]	76.6 [75.3, 77.9]	80.1 [79.0, 81.2]	83.5 [82.5, 84.5]	84.6 [83.4, 85.7]
I-XII. All tumours, malignant	74.1 [72.6, 75.7]	76.1 [74.8, 77.5]	78.9 [77.7, 80.1]	82.0 [80.9, 83.1]	83.1 [81.8, 84.3]
I. Leukaemias	75.8 [72.8, 78.7]	77.8 [75.4, 80.2]	82.1 [80.0, 84.1]	86.7 [85.0, 88.5]	86.6 [84.6, 88.7]
Ia. Lymphoid leukaemias	79.9 [76.8, 82.9]	83.0 [80.6, 85.5]	84.7 [82.6, 86.8]	89.2 [87.4, 91.0]	89.8 [87.8, 91.9]
Ib. Acute myeloid leukaemias	59.5 [51.5, 67.5]	59.3 [52.8, 65.8]	67.6 [61.3, 73.9]	75.0 [69.3, 80.6]	72.5 [65.6, 79.5]
II. Lymphomas	86.4 [83.2, 89.6]	87.7 [84.8, 90.6]	93.1 [91.0, 95.2]	94.0 [92.0, 95.9]	94.4 [92.2, 96.5]
IIa. Hodgkin lymphomas	94.6 [91.3, 97.8]	94.0 [90.5, 97.4]	97.6 [95.5, 99.7]	97.3 [95.1, 99.4]	97.8 [95.6, 99.9]
IIb. Non-Hodgkin lymphomas (except for Burkitt)	75.7 [68.6, 82.7]	78.4 [72.0, 84.7]	88.7 [84.2, 93.3]	87.0 [82.2, 91.7]	87.7 [82.4, 93.0]
IIc. Burkitt lymphoma	86.7 [80.6, 92.8]	90.0 [85.4, 94.7]	92.4 [88.4, 96.4]	97.6 [95.3, 99.9]	97.4 [94.5, 100]
III. CNS	66.6 [63.1, 70.2]	66.6 [63.5, 69.7]	70.7 [68.1, 73.3]	76.0 [73.6, 78.3]	78.2 [75.6, 80.9]
III. CNS, malignant	54.2 [49.6, 58.8]	58.6 [54.7, 62.5]	59.6 [56.2, 63.0]	63.7 [60.4, 67.1]	66.2 [62.5, 69.9]
III. CNS, non-malignant	92.3 [88.7, 95.8]	82.9 [78.7, 87.2]	94.0 [91.7, 96.4]	99.1 [98.2, 100]	99.4 [98.6, 100]
IIIa. Ependymomas and choroid plexus tumour	62.4 [52.3, 72.6]	62.2 [53.1, 71.3]	69.8 [61.9, 77.7]	74.9 [67.6, 82.2]	78.6 [70.6, 86.5]
IIIa. Ependymomas and choroid plexus tumour, malignant	53.5 [41.9, 65.1]	57.1 [47.0, 67.2]	65.3 [56.3, 74.2]	66.9 [57.6, 76.1]	72.1 [61.9, 82.3]
IIIb. Astrocytomas	79.9 [75.1, 84.7]	78.4 [73.8, 82.9]	85.3 [82.1, 88.6]	84.7 [81.7, 87.8]	88.5 [85.3, 91.7]
IIIb. Astrocytomas, malignant	68.9 [61.6, 76.2]	69.9 [63.3, 76.6]	74.1 [68.5, 79.6]	73.2 [68.2, 78.2]	77.0 [71.1, 83.0]
IIIb. Astrocytomas, non-malignant	94.7 [90.6, 98.8]	89.7 [84.5, 94.8]	98.1 [96.3, 99.9]	99.6 [98.7, 100]	100
IIIc. Intracranial and intraspinal embryonal tumours	47.5 [39.6, 55.5]	54.4 [47.7, 61.0]	49.5 [43.7, 55.3]	52.9 [46.3, 59.5]	63.6 [56.1, 71.1]
IIId. Other gliomas	44.5 [31.3, 57.8]	50.0 [40.7, 59.3]	53.8 [45.8, 61.7]	60.4 [53.1, 67.7]	50.1 [42.2, 57.9]
IV. Peripheral nervous cell tumours	72.2 [67.5, 76.9]	73.6 [69.3, 78.0]	75.7 [71.6, 79.8]	82.3 [78.6, 86.1]	85.1 [80.9, 89.3]
IVa. Neuroblastoma and ganglioneuroblastoma	72.1 [67.4, 76.8]	73.6 [69.3, 78.0]	75.5 [71.4, 79.7]	82.0 [78.2, 85.8]	84.9 [80.6, 89.2]
V. Retinoblastoma	96.9 [93.4, 100]	96.9 [93.9, 99.9]	95.4 [92.0, 98.7]	99.3 [98.0, 100]	99.0 [97.2, 100]
VI. Renal tumours	87.1 [81.6, 92.5]	89.2 [85.3, 93.0]	91.9 [88.7, 95.2]	93.9 [90.9, 96.9]	92.5 [88.8, 96.3]
VIa. Nephroblastoma	86.7 [81.1, 92.3]	89.0 [85.1, 92.9]	91.8 [88.5, 95.1]	93.7 [90.6, 96.8]	92.8 [89.0, 96.6]
VII. Hepatic tumours	69.2 [55.8, 82.7]	75.1 [64.5, 85.7]	86.1 [78.1, 94.1]	74.8 [64.5, 85.2]	79.8 [68.6, 91.0]
VIIa. Hepatoblastoma	74.4 [60.7, 88.1]	79.7 [69.4, 89.9]	86.8 [78.8, 94.9]	76.6 [65.5, 87.7]	81.1 [69.3, 92.9]
VIII. Malignant bone tumours	70.8 [65.0, 76.6]	62.0 [56.1, 67.8]	71.0 [65.8, 76.2]	67.9 [62.8, 72.9]	69.1 [63.4, 74.8]
VIIIa. Osteosarcomas	72.8 [64.2, 81.4]	63.0 [53.9, 72.0]	69.8 [61.7, 77.8]	65.8 [58.1, 73.5]	69.0 [60.4, 77.7]
VIIIc. Ewing tumour and related sarcomas of bone	68.2 [60.2, 76.3]	61.8 [54.0, 69.6]	70.5 [63.4, 77.6]	67.2 [60.0, 74.5]	66.2 [58.1, 74.3]
IX. Soft tissue sarcomas and other extraosseous sarcomas	64.0 [57.6, 70.4]	72.6 [67.1, 78.1]	69.0 [63.8, 74.3]	75.9 [71.1, 80.6]	77.5 [72.2, 82.8]
IXa. Rhabdomyosarcomas	66.5 [58.2, 74.8]	67.9 [60.1, 75.6]	67.3 [60.0, 74.7]	77.5 [70.8, 84.3]	78.8 [71.3, 86.4]
IXb + d + e. Non-rhabdomyosarcoma soft tissue sarcomas	60.5 [50.4, 70.5]	78.3 [70.8, 85.9]	70.7 [63.2, 78.1]	73.9 [67.1, 80.7]	75.8 [68.1, 83.4]
X. Germ cell tumours	84.1 [77.3, 90.8]	86.4 [80.8, 91.9]	93.4 [89.5, 97.4]	89.8 [85.3, 94.3]	96.3 [93.0, 99.5]
Xa. Intracranial and intraspinal germ cell tumours	75.7 [61.9, 89.5]	73.3 [60.4, 86.3]	93.8 [86.9, 100]	81.0 [71.3, 90.7]	92.7 [84.7, 100]
Xb. Extracranial and extragonadal germ cell tumours	80.0 [65.7, 94.3]	86.5 [75.4, 97.5]	86.7 [75.8, 97.6]	90.2 [81.0, 99.3]	93.9 [85.8, 100]
Xc. Gonadal germ cell tumours	93.3 [86.1, 100]	95.3 [90.1, 100]	98.4 [95.3, 100]	97.2 [93.3, 100]	100
XI. Epithelial neoplasms and melanomas	98.3 [94.8, 100]	90.6 [84.8, 96.5]	88.1 [82.0, 94.2]	90.7 [85.9, 95.5]	89.8 [84.1, 95.5]
XIb. Thyroid carcinomas	100	100	100	100	100

* Survival estimates for 2019–2022 were obtained using the period approach, with cases diagnosed across the period 2014–2021. Note: Number of cases by diagnosis cohort: 3236 in 1999–2003, 4218 in 2004–2008, 4959 in 2009–2013, 5098 in 2014–2018, and 3023 in 2019–2021.

**Table 3:** Observed 5-year survival by age group, 2009–2018.

Diagnostic Group	5-Year OS Rate (%) [95% CI]				*p* Log-Rank
	<1 Year	1–4 Years	5–9 Years	10–14 Years	
I-XII. All tumours	81.4 [79.1, 83.7]	83.4 [82.1, 84.6]	81.3 [79.9, 82.8]	80.5 [79.0, 82.0]	0.02
I-XII. All tumours, malignant	80.7 [78.3, 83.1]	82.4 [81.1, 83.7]	79.5 [77.9, 81.1]	78.8 [77.2, 80.5]	0.005
I. Leukaemias	60.0 [52.2, 67.8]	89.7 [88.0, 91.4]	86.3 [83.9, 88.7]	77.4 [74.1, 80.7]	<0.0001
Ia. Lymphoid leukaemias	55.8 [44.8, 66.9]	91.6 [89.9, 93.2]	88.5 [86.1, 90.9]	78.8 [75.0, 82.7]	<0.0001
Ib. Acute myeloid leukaemias	61.1 [48.9, 73.3]	74.8 [67.5, 82.2]	74.2 [66.2, 82.1]	70.3 [62.4, 78.2]	0.2
II. Lymphomas	93.5 [90.0, 97.1]		94.7 [92.5, 96.8]	92.7 [90.6, 94.9]	0.5
IIa. Hodgkin lymphomas	-		98.2 [95.8, 100]	97.0 [95.1, 98.9]	-
IIb. Non-Hodgkin lymphomas (except for Burkitt)	88.1 [81.2, 95.0]		92.3 [87.9, 96.7]	83.5 [77.6, 89.4]	0.06
IIc. Burkitt lymphoma	97.7 [94.6, 100]		94.3 [90.7, 97.9]	93.6 [88.7, 98.6]	0.4
III. CNS	63.4 [56.0, 70.8]	74.0 [71.0, 77.0]	71.1 [68.1, 74.1]	79.0 [75.6, 82.4]	<0.0001
III. CNS, malignant	47.7 [38.3, 57.1]	65.5 [61.7, 69.3]	58.1 [54.0, 62.1]	65.6 [60.4, 70.8]	<0.0001
III. CNS, non-malignant	94.6 [88.5, 100]	96.9 [94.6, 99.2]	96.1 [93.9, 98.2]	97.8 [96.0, 99.7]	0.5
IIIa. Ependymomas and choroid plexus tumour	51.5 [34.5, 68.6]	67.2 [58.7, 75.8]	81.0 [71.3, 90.7]	86.7 [77.5, 95.9]	0.0003
IIIa. Ependymomas and choroid plexus tumour, malignant	31.8 [12.4, 51.3]	63.4 [54.1, 72.7]	76.5 [64.8, 88.1]	81.3 [67.7, 94.8]	<0.0001
IIIb. Astrocytomas	81.1 [70.6, 91.7]	92.1 [89.3, 95.0]	81.9 [77.9, 85.8]	80.0 [74.6, 85.3]	0.0001
IIIb. Astrocytomas, malignant	72.7 [57.5, 87.9]	87.7 [83.2, 92.2]	65.8 [59.1, 72.5]	61.0 [51.9, 70.1]	<0.0001
IIIb. Astrocytomas, non-malignant	95.0 [85.5, 100]	98.6 [96.6, 100]	98.9 [97.4, 100]	100	0.3
IIIc. Intracr/intrasp embryonal tumours	27.5 [13.7, 41.3]	44.4 [37.4, 51.3]	56.9 [49.7, 64.1]	64.8 [54.5, 75.2]	<0.0001
IIId. Other gliomas	72.1 [63.0, 81.2]		41.5 [33.2, 49.7]	65.5 [55.9, 75.0]	<0.0001
IV. Peripheral nervous cell tumours	92.3 [89.6, 95.0]	68.1 [63.3, 73.0]	63.2 [52.0, 74.5]	86.4 [72.0, 100]	<0.0001
IVa. Neuroblastoma/ganglioneuroblastoma	92.3 [89.5, 95.0]	68.0 [63.1, 72.8]	62.1 [50.7, 73.6]	82.4 [64.2, 100]	<0.0001
V. Retinoblastoma	97.6 [94.8, 100]	97.0 [94.3, 99.6]	-	-	-
VI. Renal tumours	89.5 [83.0, 96.0]	94.7 [92.1, 97.3]	91.4 [86.6, 96.3]	91.3 [79.8, 100]	0.3
VIa. Nephroblastoma	89.5 [83.0, 96.0]	94.6 [92.0, 97.3]	91.0 [85.9, 96.1]	88.2 [72.9, 100]	0.2
VII. Hepatic tumours	78.7 [66.3, 91.1]	93.5 [87.3, 99.7]	72.2 [51.5, 92.9]	47.1 [23.3, 70.8]	0.0002
VIIa. Hepatoblastoma	77.6 [64.7, 90.5]	93.2 [86.7, 99.6]	75.0 [53.8, 96.2]	-	-
VIII. Malignant bone tumours	65.0 [50.2, 79.8]		75.0 [68.8, 81.2]	67.1 [62.4, 71.8]	0.2
VIIIa. Osteosarcomas	-		70.0 [59.8, 80.3]	67.5 [60.8, 74.2]	-
VIIIc. Ewing tumour and related sarcomas of bone	66.7 [49.8, 83.5]		76.6 [68.4, 84.8]	64.9 [58.1, 71.7]	0.1
IX. Soft tissue sarcomas and other extraosseous sarcomas	68.3 [57.1, 79.5]	74.2 [67.8, 80.6]	76.5 [69.9, 83.0]	69.3 [63.0, 75.6]	0.3
IXa. Rhabdomyosarcomas	80.0 [62.5, 97.5]	74.4 [66.8, 82.1]	73.6 [64.6, 82.7]	64.1 [52.7, 75.6]	0.3
IXb + d + e. Non-rhabdomyosarcoma STS	63.3 [49.4, 77.2]	72.0 [59.6, 84.5]	80.1 [70.7, 89.4]	71.6 [64.1, 79.2]	0.1
X. Germ cell tumours	89.4 [81.4, 97.4]	86.8 [78.2, 95.3]	91.6 [85.6, 97.5]	94.5 [90.5, 98.5]	0.3
Xa. Intracr/intrasp germ cell tumours	-		89.4 [80.6, 98.2]	90.5 [82.5, 98.4]	-
Xb. Extracranial and extragonadal germ cell tumours	89.1 [79.0, 99.2]	82.0 [67.6, 96.3]	-	-	-
Xc. Gonadal germ cell tumours	100	100	92.9 [83.3, 100]	98.5 [95.5, 100]	0.2
XI. Epithelial neoplasms and melanomas	70.4 [51.9, 88.8]		89.2 [82.0, 96.3]	92.8 [88.7, 96.9]	0.003
XIb. Thyroid carcinomas	-		100	100	-

Note: Cases younger than 5 years were pooled into a single group when either age group (0 or 1–4 years) did not reach 15 cases in the 2009–2018 cohort.

## Data Availability

The datasets generated and/or analysed during the current study are not publicly available due to privacy restrictions, but aggregated tables, as well as the R code used for the analysis, can be made available from the corresponding authors upon reasonable request.

## Supplementary Materials

The following supporting information can be downloaded from https://www.mdpi.com/article/10.3390/cancers18030362/s1. Table S1. Age-specific case population weights sampled from the study case pool, 1999–2021. Table S2. Registration quality indicators by major diagnostic group, 1999–2021. Table S3. Survival results by incidence cohort for all tumours, 0–14 years. Table S4. Survival results by incidence cohort for haematological neoplasms, 0–14 years. Table S5. Survival results by incidence cohort for central nervous system tumours, 0–14 years. Table S6. Survival results by incidence cohort for embryonal and germ cell tumours, 0–14 years. Table S7. Survival results by incidence cohort for sarcomas and epithelial tumours, 0–14 years. Table S8. Number of registered cases by age group in the 2009–2018 incidence cohort by diagnostic group. Table S9. Observed survival at 5 years by sex and diagnostic group in the 2009–2018 incidence cohort, 0–14 years. Table S10. Age-standardised overall survival rates (StOS) by diagnostic group and incidence cohort. Table S11. Number of recorded epithelial tumour cases by incidence cohort, 0–14 years.

## Abbreviations

The following abbreviations are used in this manuscript:

AACS	Absolute annual change in survival
ALCL	Anaplastic large cell lymphoma
AML	Acute myeloid leukaemia
BIC	Bayesian information criterion
BL	Burkitt lymphoma
CI	Confidence interval
CNS	Central nervous system
GCT	Germ cell tumours
HIC	High-income countries
HL	Hodgkin lymphoma
ICCC	International Childhood Cancer Classification
ICD-O	International Classification of Diseases for Oncology
LBL	Lymphoblastic lymphoma
LL	Lymphoid leukaemias
NHL	Non-Hodgkin lymphomas
OS	Overall survival
RETI-SEHOP	Spanish Registry of Childhood Tumours
SEHOP	Spanish Society of Paediatric Haematology and Oncology

## Appendix A

Members of the RETI-SEHOP Survival Working Group are disclosed in Appendix A.

RETI-SEHOP Survival Working Group: Spanish Registry of Childhood Tumours (RETI-SEHOP) central team, University of Valencia: A Cañete Nieto, R Peris-Bonet, E Pardo Romaguera, P Alfonso-Comos, S Porta Cebolla, S Valero Poveda. WG-RETI of the Spanish Society of Paediatric Haematology and Oncology (SEHOP): O Cruz Martínez (Sant Joan de Déu Hospital, Barcelona), A Fernández-Teijeiro (Virgen de la Macarena University Hospital, Seville), L Gros Subias (Vall d’Hebron Hospital, Barcelona), I Gutiérrez Carrasco (Virgen del Rocío Hospital, Seville), R López Almaraz (Cruces University Hospital, Barakaldo), B Martínez de las Heras (La Fe Hospital, Valencia), A Sastre Urgelles (La Paz Hospital, Madrid), M Tasso Cereceda (Doctor Balmis General University Hospital, Alicante). SEHOP reporting centres registration co-ordinators: F Lendínez Molinos (Torrecárdenas Hospital, Almería); J Morán Sánchez (Puerta del Mar Hospital, Cádiz); ME Mateos González (Reina Sofía Hospital, Córdoba); MJ Ortega Acosta (Virgen de las Nieves Hospital, Granada); AI González Espín (Maternity Hospital, Jaén); EM Gálvez de la Villa (University Hospital, Jerez de la Frontera); G Gutiérrez Schiaffino, MR Prieto Bonilla (Regional University Hospital, Málaga); A Fernández-Teijeiro Álvarez (Virgen de la Macarena University Hospital, Seville); C Márquez Vega, JM Pérez-Hurtado de Mendoza (Virgen del Rocío Hospital, Seville); A Muñoz Mellado (Miguel Servet Hospital, Zaragoza); JA Villegas Rubio, S González Muñiz (Asturias Central Hospital, Oviedo); JA Salinas Sanz (Son Espases Hospital, Palma de Mallorca); K Melwani Melwani, A Molinés Honrubia (Canary Islands Maternity Hospital, Las Palmas); J Gómez Sirvent (Nuestra Señora de la Candelaria Hospital, Santa Cruz de Tenerife); M González Cruz (Canary Islands University Hospital, Santa Cruz de Tenerife); M López Duarte (Marqués de Valdecilla University Hospital, Santander); MI Buedo Rubio (General University Hospital, Albacete); M Zamora Gómez (University Hospital, Toledo); S López Iniesta (University Hospital, León); R Portugal Rodríguez (University Hospital, Burgos); MC Mendoza Sánchez (Clinical University Hospital, Salamanca); H González García (Clinical University Hospital, Valladolid); M Gorostegui Obanos (Sant Joan de Déu Hospital, Barcelona); M Torrent Español (Sant Pau Hospital, Barcelona); L Moreno Martín Retortillo (Vall d’Hebron Hospital, Barcelona); M Tasso Cereceda (Doctor Balmis General University Hospital, Alicante); C Nova Lozano (Clinical University Hospital, Valencia); N Benavent (La Fe Hospital, Valencia); R López Almaraz (Cruces University Hospital, Barakaldo); M Abós García (Donostia University Hospital, San Sebastián); M Mora Matilla, JM Vagace Valero (Maternity Hospital, Badajoz); GM Muñoz García (Coruña University Hospital (CHUAC), A Coruña); M Fernández Sanmartín (Clinical University Hospital, Santiago de Compostela); M Tallón García (Álvaro Cunqueiro Hospital, Vigo); M Baro Fernández (12 de Octubre Hospital, Madrid); EJ Bardón Cancho (Gregorio Marañón Hospital, Madrid); A Sastre Urgelles (La Paz Hospital, Madrid); A Lassaletta Atienza (Niño Jesús Hospital, Madrid); B Herrero Velasco (Sanitas Hospital, La Moraleja, Madrid); B López-Ibor Aliño (Montepríncipe Hospital, Madrid); L Madero López (Quirónsalud Hospital, Madrid); JL Fuster Soler (Virgen de la Arrixaca Hospital, Murcia); E Panizo Morgado (Navarre Clinical University Hospital, Pamplona); M Oscoz Lizarbe (Navarre University Hospital, Pamplona). SEHOP executive board: A Cañete Nieto (La Fe Hospital, Valencia), JL Dapena Díaz (Sant Joan de Déu Hospital, Barcelona), MA García-Ariza (Cruces University Hospital, Barakaldo), I Jiménez García (Virgen de la Arrixaca Hospital, Murcia), M Mora Matilla (Maternity Hospital, Badajoz), Alba Rubio-San-Simón (Niño Jesús Hospital, Madrid), María Tallón García (Álvaro Cunqueiro Hospital, Vigo).
