# Supplementary material for: Spanish Paediatric Haematology and Oncology Survival Results and Trends, 1999–2022
Source: Cancers (Basel). 2026 Jan 23;18(3):362. doi: 10.3390/cancers18030362 (PMC12897301; doi:10.3390/cancers18030362)
Supplement: Supplementary file 1 [file cancers-18-00362-s001.zip › cancers-4098339-supplementary.pdf]

Supplementary Material

Table of Contents

Table S1. Age-specific case population weights sampled from the study case pool, 1999–2021. 2

Table S2. Registration quality indicators by major diagnostic group, 1999–2021. 3

Table S3. Survival results by incidence cohort for all tumours, 0–14 years. 4

Table S4. Survival results by incidence cohort for haematological neoplasms, 0–14 years. 4

Table S5. Survival results by incidence cohort for central nervous system tumours, 0–14 years. 5

Table S6. Survival results by incidence cohort for embryonal and germ cell tumours, 0–14 years. 6

Table S7. Survival results by incidence cohort for sarcomas and epithelial tumours, 0–14 years. 8

Table S8. Number of registered cases by age group in the 2009–2018 incidence cohort by diagnostic group. 9

Table S9. Observed survival at 5 years by sex and diagnostic group in the 2009–2018 incidence cohort, 0–14 years. 10

Table S10. Age-standardised overall survival rates (StOS) by diagnostic group and incidence cohort. 11

Table S11. Number of recorded epithelial tumour cases by incidence cohort, 0–14 years. 13

Table S1. Age-specific case population weights sampled from the study case pool, 1999–2021.

Diagnostic group	No. cases (%)			Total
	0–4 years	5–9 years	10–14 years	
**I-XII. All tumours**	**9,227 (44.9%)**	**5,722 (27.9%)**	**5,585 (27.2%)**	**20,534**
**I-XII. All tumours, malignant**	**8,679 (46.0%)**	**5,109 (27.1%)**	**5,089 (27.0%)**	**18,877**
I. Leukaemias	2,789 (49.8%)	1,624 (29.0%)	1,183 (21.1%)	5,596
Ia. Lymphoid leukaemias	2,319 (51.5%)	1,341 (29.8%)	845 (18.8%)	4,505
Ib. Acute myeloid leukaemias	414 (44.7%)	241 (26.0%)	272 (29.3%)	927
II. Lymphomas	385 (15.5%)	886 (35.7%)	1,210 (48.8%)	2,481
IIa. Hodgkin lymphomas	286 (30.1%)		665 (69.9%)	951
IIb. Non-Hodgkin lymphomas (except Burkitt)	161 (19.8%)	304 (37.3%)	350 (42.9%)	815
IIc. Burkitt lymphoma	180 (25.2%)	340 (47.7%)	193 (27.1%)	713
III. CNS	1,922 (39.6%)	1,731 (35.7%)	1,196 (24.7%)	4,849
III. CNS, malignant	1,379 (42.9%)	1,128 (35.1%)	705 (21.9%)	3,212
III. CNS, non-malignant	543 (33.2%)	603 (36.8%)	491 (30.0%)	1,637
IIIa. Ependymomas and choroid plexus tumour	327 (59.5%)	223 (40.5%)		550
IIIa. Ependymomas and choroid plexus tumour, malignant	271 (61.5%)	170 (38.5%)		441
IIIb. Astrocytomas	733 (38.6%)	704 (37.1%)	461 (24.3%)	1,898
IIIb. Astrocytomas, malignant	425 (40.9%)	370 (35.6%)	245 (23.6%)	1,040
IIIb. Astrocytomas, non-malignant	308 (35.9%)	334 (38.9%)	216 (25.2%)	858
IIIc. Intracranial and intraspinal embryonal tumours	469 (45.9%)	366 (35.8%)	187 (18.3%)	1,022
IIId. Other gliomas	180 (28.9%)	255 (41.0%)	187 (30.1%)	622
IV. Peripheral nervous cell tumours	1,565 (87.7%)	220 (12.3%)		1,785
IVa. Neuroblastoma and ganglioneuroblastoma	1,558 (88.2%)	208 (11.8%)		1,766
V. Retinoblastoma	608 (100%)			608
VI. Renal tumours	787 (73.7%)	281 (26.3)		1,068
VIa. Nephroblastoma	783 (75.1%)	259 (24.9%)		1,042
VII. Hepatic tumours	293 (100%)			293
VIIa. Hepatoblastoma	261 (100%)			261
VIII. Malignant bone tumours	98 (7.4%)	383 (28.9%)	846 (63.8%)	1,327
VIIIa. Osteosarcomas	171 (29.8%)		402 (70.2%)	573
VIIIc. Ewing tumour and related sarcomas of bone	287 (40.7%)		419 (59.3%)	706
IX. Soft tissue sarcomas and other extraosseous sarcomas	511 (40.1%)	355 (27.9%)	408 (32.0%)	1,274
IXa. Rhabdomyosarcomas	324 (48.9%)	208 (31.4%)	131 (19.8%)	663
IXb+d+e. Non-rhabdomyosarcoma soft tissue sarcomas	184 (30.3%)	147 (24.2%)	276 (45.5%)	607
X. Germ cell tumours	271 (38.5%)	146 (20.8%)	286 (40.7%)	703
Xa. Intracranial and intraspinal germ cell tumours	108 (48.0%)		117 (52.0%)	225
Xb. Extracranial and extragonadal germ cell tumours	171 (100%)			171
Xc. Gonadal germ cell tumours	141 (48.3%)		151 (51.7%)	292
XI. Epithelial neoplasms and melanomas	192 (37.6%)		318 (62.4%)	510
XIb. Thyroid carcinomas	158 (100%)			158

Table S2. Registration quality indicators by major diagnostic group, 1999–2021.

Diagnostic group	MV (%)	NOS (%)*	<1 year (%)	Non-malignant (%)
I. Leukaemias	99.8	0.04	5.2	-
II. Lymphomas	99.8	0.00	0.6	-
III. CNS	77.3	2.66	6.9	33.8
IV. Peripheral nervous cell tumours	90.3	-	43.5	-
V. Retinoblastoma	27.0	-	42.4	-
VI. Renal tumours	79.7	0.19	17.3	-
VII. Hepatic tumours	90.8	0.00	29.4	-
VIII. Malignant bone tumours	97.4	0.60	0.6	-
IX. Soft tissue and other extraosseous sarcomas	96.7	6.04	9.8	-
X. Germ cell tumours	89.6	1.14	17.8	2.8†
XI. Epithelial neoplasms and melanomas	95.9	37.84	2.2	-
XII. Other and unspecified malignant neoplasms	95.0	5.00	25.0	-

* Percentage of cases classified as IIe, IIIf, VIc, VIIc, VIIIe, IXe, Xe, XIf and XIIb, and leukaemias with histology codes 8000, 8800 and 9800.

† Non-malignant extracranial/extraspinal germ cell tumours are not registered. Among CNS germ cell tumours (Xa), 8.9% are non-malignant, corresponding to an overall non-malignant percentage of 2.8% for group X.

Table S3. Survival results by incidence cohort for all tumours, 0–14 years. OS rates for 2019–2022 were estimated following the period approach.

Diagnostic group	Incidence cohort	No.	5y FU (%)	1y OS (%) [95% CI]	3y OS (%) [95% CI]	5y OS (%) [95% CI]
I-XII. All tumours	1999–2003	3,236	97.5	87.7 [86.6, 88.9]	78.4 [77.0, 79.8]	75.4 [73.9, 76.9]
	2004–2008	4,218	96.4	89.9 [89.0, 90.8]	79.5 [78.3, 80.7]	76.6 [75.3, 77.9]
	2009–2013	4,959	96.4	91.6 [90.8, 92.4]	83.0 [81.9, 84.0]	80.1 [79.0, 81.2]
	2014–2018	5,098	96.7	93.0 [92.3, 93.7]	86.0 [85.0, 87.0]	83.5 [82.5, 84.5]
	2019–2022	7,473*	97.2	94.2 [93.4, 95.0]	87.2 [86.1, 88.3]	84.6 [83.4, 85.7]
I-XII. All tumours, malignant	1999–2003	3,010	97.8	87.1 [85.9, 88.3]	77.3 [75.8, 78.8]	74.1 [72.6, 75.7]
	2004–2008	3,910	96.6	89.7 [88.8, 90.7]	79.1 [77.8, 80.4]	76.1 [74.8, 77.5]
	2009–2013	4,565	96.5	91.2 [90.4, 92.0]	82.0 [80.9, 83.1]	78.9 [77.7, 80.1]
	2014–2018	4,658	97.0	92.3 [91.6, 93.1]	84.7 [83.7, 85.8]	82.0 [80.9, 83.1]
	2019–2022	6,753*	97.6	93.6 [92.8, 94.5]	85.9 [84.8, 87.1]	83.1 [81.8, 84.3]

* The number of contributing cases for period survival are the number of patients diagnosed in 2014–2021 with survival experience during 2019–2022 up to 5 years after diagnosis.

Table S4. Survival results by incidence cohort for haematological neoplasms, 0–14 years. OS rates for 2019–2022 were estimated following the period approach.

Diagnostic group	Incidence cohort	No.	5y FU (%)	1y OS (%) [95% CI]	3y OS (%) [95% CI]	5y OS (%) [95% CI]
**I. Leukaemias**	**1999–2003**	**822**	**98.3**	**86.9 [84.6, 89.3]**	**78.4 [75.5, 81.2]**	**75.8 [72.8, 78.7]**
	**2004–2008**	**1,146**	**98.3**	**89.9 [88.2, 91.7]**	**80.2 [77.9, 82.5]**	**77.8 [75.4, 80.2]**
	**2009–2013**	**1,388**	**97.3**	**92.1 [90.7, 93.6]**	**84.8 [82.9, 86.7]**	**82.1 [80.0, 84.1]**
	**2014–2018**	**1,428**	**97.1**	**94.2 [93.0, 95.4]**	**89.0 [87.4, 90.6]**	**86.7 [85.0, 88.5]**
	**2019–2022**	**2,094***	**98.3**	**94.8 [93.4, 96.2]**	**88.9 [86.9, 90.8]**	**86.6 [84.6, 88.7]**
Ia. Lymphoid leukaemias	1999–2003	659	98.3	90.4 [88.2, 92.7]	82.5 [79.6, 85.4]	79.9 [76.8, 82.9]
	2004–2008	895	98.2	93.6 [92.0, 95.2]	85.6 [83.3, 87.9]	83.0 [80.6, 85.5]
	2009–2013	1,133	97.6	94.3 [93.0, 95.7]	87.3 [85.4, 89.3]	84.7 [82.6, 86.8]
	2014–2018	1,148	97.5	95.7 [94.6, 96.9]	91.5 [89.9, 93.1]	89.2 [87.4, 91.0]
	2019–2022	1,726*	98.4	96.1 [94.8, 97.5]	91.9 [90.1, 93.8]	89.8 [87.8, 91.9]
Ib. Acute myeloid leukaemias	1999–2003	144	99.3	72.1 [64.8, 79.4]	62.3 [54.4, 70.2]	59.5 [51.5, 67.5]
	2004–2008	222	98.2	77.0 [71.4, 82.5]	61.1 [54.7, 67.6]	59.3 [52.8, 65.8]
	2009–2013	214	96.3	81.3 [76.1, 86.5]	71.0 [64.9, 77.1]	67.6 [61.3, 73.9]
	2014–2018	232	95.3	86.5 [82.1, 90.9]	77.2 [71.8, 82.7]	75.0 [69.3, 80.6]
	2019–2022	297*	98.7	88.4 [83.1, 93.8]	74.8 [68.0, 81.6]	72.5 [65.6, 79.5]
**II. Lymphomas**	**1999–2003**	**452**	**98.0**	**92.0 [89.5, 94.5]**	**87.5 [84.5, 90.6]**	**86.4 [83.2, 89.6]**
	**2004–2008**	**506**	**97.0**	**92.1 [89.7, 94.4]**	**88.3 [85.5, 91.1]**	**87.7 [84.8, 90.6]**
	**2009–2013**	**571**	**96.2**	**96.3 [94.8, 97.9]**	**93.7 [91.7, 95.7]**	**93.1 [91.0, 95.2]**
	**2014–2018**	**585**	**97.4**	**96.4 [94.9, 97.9]**	**94.9 [93.1, 96.6]**	**94.0 [92.0, 95.9]**
	**2019–2022**	**918***	**98.0**	**97.1 [95.6, 98.7]**	**95.5 [93.5, 97.4]**	**94.4 [92.2, 96.5]**
IIa. Hodgkin lymphomas	1999–2003	185	97.8	99.5 [98.4, 100]	96.2 [93.4, 99.0]	94.6 [91.3, 97.8]
	2004–2008	183	98.9	98.9 [97.4, 100]	95.6 [92.6, 98.6]	94.0 [90.5, 97.4]
	2009–2013	212	95.8	98.6 [97.0, 100]	97.6 [95.5, 99.7]	97.6 [95.5, 99.7]
	2014–2018	223	97.3	99.1 [97.9, 100]	99.1 [97.9, 100]	97.3 [95.1, 99.4]
	2019–2022	366*	97.8	100	100	97.8 [95.6, 99.9]

Table S4 (continued)

Diagnostic group	Incidence cohort	No.	5y FU (%)	1y OS (%) [95% CI]	3y OS (%) [95% CI]	5y OS (%) [95% CI]
IIb. Non-Hodgkin lymphomas (except Burkitt)	1999–2003	146	96.6	85.5 [79.7, 91.2]	77.1 [70.2, 83.9]	75.7 [68.6, 82.7]
	2004–2008	162	97.5	84.0 [78.3, 89.6]	78.4 [72.0, 84.7]	78.4 [72.0, 84.7]
	2009–2013	188	96.8	95.2 [92.1, 98.3]	89.3 [84.9, 93.7]	88.7 [84.2, 93.3]
	2014–2018	192	97.9	92.2 [88.4, 96.0]	87.5 [82.8, 92.2]	87.0 [82.2, 91.7]
	2019–2022	297*	97.3	92.9 [88.7, 97.2]	88.4 [83.2, 93.6]	87.7 [82.4, 93.0]
IIb. Burkitt lymphoma	1999–2003	120	100.0	88.3 [82.6, 94.1]	86.7 [80.6, 92.8]	86.7 [80.6, 92.8]
	2004–2008	161	94.4	92.6 [88.5, 96.6]	90.0 [85.4, 94.7]	90.0 [85.4, 94.7]
	2009–2013	171	95.9	94.7 [91.4, 98.1]	93.6 [89.9, 97.2]	92.4 [88.4, 96.4]
	2014–2018	169	97.0	97.6 [95.3, 99.9]	97.6 [95.3, 99.9]	97.6 [95.3, 99.9]
	2019–2022	254*	99.2	98.2 [95.7, 100]	97.4 [94.5, 100]	97.4 [94.5, 100]

* The number of contributing cases for period survival are the number of patients diagnosed in 2014–2021 with survival experience during 2019–2022 up to 5 years after diagnosis.

Table S5. Survival results by incidence cohort for central nervous system tumours, 0–14 years. OS rates for 2019–2022 were estimated following the period approach.

Diagnostic group	Incidence cohort	No.	5y FU (%)	1y OS (%) [95% CI]	3y OS (%) [95% CI]	5y OS (%) [95% CI]
**III. CNS**	**1999–2003**	**679**	**96.8**	**81.3 [78.4, 84.3]**	**68.9 [65.4, 72.4]**	**66.6 [63.1, 70.2]**
	**2004–2008**	**930**	**95.6**	**84.0 [81.6, 86.3]**	**69.8 [66.9, 72.8]**	**66.6 [63.5, 69.7]**
	**2009–2013**	**1,200**	**96.8**	**85.4 [83.4, 87.4]**	**73.9 [71.4, 76.4]**	**70.7 [68.1, 73.3]**
	**2014–2018**	**1,250**	**96.5**	**88.2 [86.4, 90.0]**	**78.8 [76.6, 81.1]**	**76.0 [73.6, 78.3]**
	**2019–2022**	**1,797***	**96.0**	**90.9 [89.1, 92.8]**	**81.2 [78.7, 83.7]**	**78.2 [75.6, 80.9]**
III. CNS, malignant	1999–2003	456	98.5	74.2 [70.1, 78.2]	57.3 [52.7, 61.8]	54.2 [49.6, 58.8]
	2004–2008	627	96.2	80.2 [77.0, 83.3]	62.8 [59.0, 66.6]	58.6 [54.7, 62.5]
	2009–2013	808	98.0	80.3 [77.5, 83.0]	64.1 [60.8, 67.4]	59.6 [56.2, 63.0]
	2014–2018	815	97.8	82.2 [79.6, 84.8]	68.0 [64.8, 71.2]	63.7 [60.4, 67.1]
	2019–2022	1,087*	97.5	85.9 [83.1, 88.8]	70.6 [67.0, 74.2]	66.2 [62.5, 69.9]
III. CNS, non-malignant	1999–2003	223	93.3	95.9 [93.3, 98.5]	92.7 [89.3, 96.2]	92.3 [88.7, 95.8]
	2004–2008	303	94.4	91.7 [88.6, 94.8]	84.3 [80.2, 88.5]	82.9 [78.7, 87.2]
	2009–2013	392	94.1	96.1 [94.2, 98.1]	94.3 [92.0, 96.6]	94.0 [91.7, 96.4]
	2014–2018	435	94.0	99.5 [98.9, 100]	99.3 [98.5, 100]	99.1 [98.2, 100]
	2019–2022	710*	93.7	99.7 [99.1, 100]	99.7 [99.1, 100]	99.4 [98.6, 100]
IIIa. Ependymomas and choroid plexus tumour	1999–2003	88	98.9	84.1 [76.5, 91.7]	67.1 [57.2, 76.9]	62.4 [52.3, 72.6]
	2004–2008	112	94.6	82.7 [75.6, 89.8]	68.8 [60.0, 77.5]	62.2 [53.1, 71.3]
	2009–2013	130	98.5	88.5 [83.0, 94.0]	76.9 [69.6, 84.1]	69.8 [61.9, 77.7]
	2014–2018	136	99.3	95.6 [92.1, 99.0]	83.1 [76.8, 89.4]	74.9 [67.6, 82.2]
	2019–2022	201*	97.5	94.0 [89.4, 98.7]	86.6 [80.1, 93.1]	78.6 [70.6, 86.5]
IIIa. Ependymomas and choroid plexus tumour, malignant	1999–2003	71	100.0	80.3 [71.0, 89.5]	59.2 [47.7, 70.6]	53.5 [41.9, 65.1]
	2004–2008	96	95.8	80.8 [72.9, 88.8]	64.7 [55.0, 74.4]	57.1 [47.0, 67.2]
	2009–2013	110	99.1	87.3 [81.0, 93.5]	73.5 [65.3, 81.8]	65.3 [56.3, 74.2]
	2014–2018	100	99.0	94.0 [89.4, 98.7]	78.0 [69.9, 86.1]	66.9 [57.6, 76.1]
	2019–2022	146*	98.6	93.3 [87.6, 99.0]	83.2 [74.8, 91.5]	72.1 [61.9, 82.3]
IIIb. Astrocytomas	1999–2003	273	96.7	89.2 [85.5, 92.9]	80.7 [76.0, 85.4]	79.9 [75.1, 84.7]
	2004–2008	326	95.1	91.3 [88.3, 94.4]	80.0 [75.6, 84.4]	78.4 [73.8, 82.9]
	2009–2013	453	96.7	92.7 [90.3, 95.1]	86.5 [83.3, 89.6]	85.3 [82.1, 88.6]
	2014–2018	538	95.5	91.3 [88.9, 93.6]	85.3 [82.3, 88.3]	84.7 [81.7, 87.8]
	2019–2022	777*	94.3	94.7 [92.3, 97.0]	89.5 [86.4, 92.6]	88.5 [85.3, 91.7]

Table S5 (continued)

Diagnostic group	Incidence cohort	No.	5y FU (%)	1y OS (%) [95% CI]	3y OS (%) [95% CI]	5y OS (%) [95% CI]
IIIb. Astrocytomas, malignant	1999–2003	156	98.7	81.9 [75.8, 87.9]	69.5 [62.3, 76.8]	68.9 [61.6, 76.2]
	2004–2008	188	95.2	87.6 [82.9, 92.4]	71.6 [65.1, 78.2]	69.9 [63.3, 76.6]
	2009–2013	240	97.1	87.5 [83.3, 91.7]	75.8 [70.3, 81.2]	74.1 [68.5, 79.6]
	2014–2018	303	97.4	84.8 [80.8, 88.8]	74.2 [69.2, 79.1]	73.2 [68.2, 78.2]
	2019–2022	389*	95.1	89.0 [84.4, 93.7]	78.8 [73.0, 84.6]	77.0 [71.1, 83.0]
IIIb. Astrocytomas, non-malignant	1999–2003	117	94.0	99.1 [97.4, 100]	95.7 [91.9, 99.4]	94.7 [90.6, 98.8]
	2004–2008	138	94.9	96.4 [93.2, 99.5]	91.2 [86.4, 96.0]	89.7 [84.5, 94.8]
	2009–2013	213	96.2	98.6 [97.0, 100]	98.6 [97.0, 100]	98.1 [96.3, 99.9]
	2014–2018	235	93.2	99.6 [98.7, 100]	99.6 [98.7, 100]	99.6 [98.7, 100]
	2019–2022	388*	93.6	100	100	100
IIIc. Intracranial and intraspinal embryonal tumours	1999–2003	154	98.7	70.6 [63.4, 77.8]	52.8 [44.9, 60.7]	47.5 [39.6, 55.5]
	2004–2008	216	97.7	76.8 [71.2, 82.5]	60.0 [53.4, 66.5]	54.4 [47.7, 61.0]
	2009–2013	284	99.3	77.5 [72.6, 82.3]	56.3 [50.5, 62.0]	49.5 [43.7, 55.3]
	2014–2018	219	99.1	77.6 [72.1, 83.2]	60.7 [54.3, 67.2]	52.9 [46.3, 59.5]
	2019–2022	287*	99.3	88.5 [83.7, 93.4]	72.0 [65.3, 78.8]	63.6 [56.1, 71.1]
IIId. Other gliomas	1999–2003	55	94.6	61.3 [48.4, 74.3]	44.5 [31.3, 57.8]	44.5 [31.3, 57.8]
	2004–2008	115	95.7	73.4 [65.2, 81.5]	51.8 [42.6, 61.1]	50.0 [40.7, 59.3]
	2009–2013	153	96.1	71.1 [63.9, 78.3]	56.5 [48.6, 64.4]	53.8 [45.8, 61.7]
	2014–2018	175	96.6	76.0 [69.7, 82.3]	61.6 [54.4, 68.8]	60.4 [53.1, 67.7]
	2019–2022	239*	98.3	74.8 [67.7, 81.9]	51.8 [44.0, 59.6]	50.1 [42.2, 57.9]

* The number of contributing cases for period survival are the number of patients diagnosed in 2014–2021 with survival experience during 2019–2022 up to 5 years after diagnosis.

Table S6. Survival results by incidence cohort for embryonal and germ cell tumours. OS rates for 2019–2022 were estimated following the period approach.

Diagnostic group	Incidence cohort	No.	5y FU (%)	1y OS (%) [95% CI]	3y OS (%) [95% CI]	5y OS (%) [95% CI]
**IV. Peripheral nervous cell tumours**	**1999–2003**	**353**	**97.7**	**88.1 [84.7, 91.5]**	**77.8 [73.4, 82.1]**	**72.2 [67.5, 76.9]**
	**2004–2008**	**402**	**97.0**	**90.8 [87.9, 93.6]**	**78.5 [74.5, 82.5]**	**73.6 [69.3, 78.0]**
	**2009–2013**	**420**	**96.7**	**93.6 [91.2, 95.9]**	**80.3 [76.5, 84.1]**	**75.7 [71.6, 79.8]**
	**2014–2018**	**399**	**95.5**	**93.7 [91.4, 96.1]**	**85.2 [81.7, 88.7]**	**82.3 [78.6, 86.1]**
	**2019–2022**	**557***	**95.2**	**94.3 [91.4, 97.2]**	**87.8 [83.9, 91.7]**	**85.1 [80.9, 89.3]**
IVa. Neuroblastoma and ganglioneuro-blastoma	1999–2003	351	98.3	88.0 [84.6, 91.4]	77.6 [73.2, 82.0]	72.1 [67.4, 76.8]
	2004–2008	398	97.0	90.9 [88.1, 93.8]	78.5 [74.5, 82.6]	73.6 [69.3, 78.0]
	2009–2013	417	96.6	93.5 [91.2, 95.9]	80.2 [76.3, 84.0]	75.5 [71.4, 79.7]
	2014–2018	392	95.4	93.6 [91.2, 96.0]	84.9 [81.4, 88.5]	82.0 [78.2, 85.8]
	2019–2022	547*	95.1	94.2 [91.3, 97.2]	87.6 [83.7, 91.6]	84.9 [80.6, 89.2]
**V. Retinoblastoma**	**1999–2003**	**100**	**95.0**	**97.9 [95.1, 100]**	**96.9 [93.4, 100]**	**96.9 [93.4, 100]**
	**2004–2008**	**133**	**96.2**	**98.5 [96.3, 100]**	**96.9 [93.9, 99.9]**	**96.9 [93.9, 99.9]**
	**2009–2013**	**152**	**96.7**	**99.3 [98.1, 100]**	**96.7 [93.9, 99.6]**	**95.4 [92.0, 98.7]**
	**2014–2018**	**149**	**94.0**	**100**	**99.3 [98.0, 100]**	**99.3 [98.0, 100]**
	**2019–2022**	**218***	**97.7**	**100**	**99.0 [97.2, 100]**	**99.0 [97.2, 100]**
**VI. Renal tumours**	**1999–2003**	**150**	**97.3**	**94.6 [91.0, 98.2]**	**89.1 [84.1, 94.2]**	**87.1 [81.6, 92.5]**
	**2004–2008**	**252**	**96.0**	**95.6 [93.1, 98.2]**	**90.4 [86.7, 94.0]**	**89.2 [85.3, 93.0]**
	**2009–2013**	**273**	**97.8**	**96.0 [93.6, 98.3]**	**93.0 [90.0, 96.1]**	**91.9 [88.7, 95.2]**
	**2014–2018**	**249**	**96.8**	**98.0 [96.2, 99.7]**	**94.3 [91.4, 97.2]**	**93.9 [90.9, 96.9]**
	**2019–2022**	**378***	**98.7**	**98.3 [96.3, 100]**	**93.0 [89.4, 96.7]**	**92.5 [88.8, 96.3]**

Table S6 (continued)

Diagnostic group	Incidence cohort	No.	5y FU (%)	1y OS (%) [95% CI]	3y OS (%) [95% CI]	5y OS (%) [95% CI]
VIa. Nephroblastoma	1999–2003	146	97.3	94.5 [90.7, 98.2]	88.8 [83.7, 94.0]	86.7 [81.1, 92.3]
	2004–2008	248	96.0	95.5 [93.0, 98.1]	90.2 [86.5, 93.9]	89.0 [85.1, 92.9]
	2009–2013	268	97.8	95.9 [93.5, 98.3]	92.9 [89.8, 96.0]	91.8 [88.5, 95.1]
	2014–2018	240	96.7	97.9 [96.1, 99.7]	94.1 [91.1, 97.1]	93.7 [90.6, 96.8]
	2019–2022	365*	98.6	98.2 [96.2, 100]	93.3 [89.7, 97.0]	92.8 [89.0, 96.6]
**VII. Hepatic tumours**	**1999–2003**	**46**	**97.8**	**80.4 [68.9, 91.9]**	**73.7 [60.9, 86.5]**	**69.2 [55.8, 82.7]**
	**2004–2008**	**65**	**96.9**	**84.4 [75.6, 93.3]**	**76.6 [66.3, 87.0]**	**75.1 [64.5, 85.7]**
	**2009–2013**	**74**	**91.9**	**91.8 [85.5, 98.1]**	**87.6 [80.0, 95.2]**	**86.1 [78.1, 94.1]**
	**2014–2018**	**68**	**98.5**	**85.3 [76.9, 93.7]**	**79.3 [69.7, 89.0]**	**74.8 [64.5, 85.2]**
	**2019–2022**	**96***	**96.9**	**89.4 [80.6, 98.2]**	**83.7 [73.3, 94.0]**	**79.8 [68.6, 91.0]**
VIIa. Hepatoblastoma	1999–2003	39	100.0	82.1 [70.0, 94.1]	76.9 [63.7, 90.2]	74.4 [60.7, 88.1]
	2004–2008	59	98.3	86.4 [77.7, 95.2]	79.7 [69.4, 89.9]	79.7 [69.4, 89.9]
	2009–2013	70	91.4	91.3 [84.7, 98.0]	86.8 [78.8, 94.9]	86.8 [78.8, 94.9]
	2014–2018	56	98.2	87.5 [78.8, 96.2]	82.1 [72.0, 92.1]	76.6 [65.5, 87.7]
	2019–2022	84*	96.4	92.9 [85.1, 100]	85.8 [75.3, 96.3]	81.1 [69.3, 92.9]
**X. Germ cell tumours**	**1999–2003**	**113**	**98.2**	**89.4 [83.7, 95.1]**	**86.7 [80.5, 93.0]**	**84.1 [77.3, 90.8]**
	**2004–2008**	**150**	**92.0**	**94.6 [90.9, 98.2]**	**88.4 [83.3, 93.6]**	**86.4 [80.8, 91.9]**
	**2009–2013**	**155**	**91.0**	**96.1 [93.0, 99.2]**	**94.1 [90.3, 97.8]**	**93.4 [89.5, 97.4]**
	**2014–2018**	**178**	**97.2**	**97.2 [94.7, 99.6]**	**92.1 [88.1, 96.1]**	**89.8 [85.3, 94.3]**
	**2019–2022**	**268***	**97.0**	**99.2 [97.6, 100]**	**97.8 [95.4, 100]**	**96.3 [93.0, 99.5]**
Xa. Intracranial and intraspinal germ cell tumours	1999–2003	37	100.0	83.8 [71.9, 95.7]	83.8 [71.9, 95.7]	75.7 [61.9, 89.5]
	2004–2008	45	95.6	88.9 [79.7, 98.1]	80.0 [68.3, 91.7]	73.3 [60.4, 86.3]
	2009–2013	48	93.8	97.9 [93.9, 100]	93.8 [86.9, 100]	93.8 [86.9, 100]
	2014–2018	64	98.4	93.7 [87.7, 99.7]	84.2 [75.2, 93.2]	81.0 [71.3, 90.7]
	2019–2022	83*	98.8	97.3 [92.0, 100]	95.0 [88.2, 100]	92.7 [84.7, 100]
Xb. Extracranial and extragonadal germ cell tumours	1999–2003	30	100.0	83.3 [70.0, 96.7]	80.0 [65.7, 94.3]	80.0 [65.7, 94.3]
	2004–2008	38	94.7	92.0 [83.2, 100]	86.5 [75.4, 97.5]	86.5 [75.4, 97.5]
	2009–2013	38	94.7	89.5 [79.7, 99.2]	89.5 [79.7, 99.2]	86.7 [75.8, 97.6]
	2014–2018	41	97.6	100	95.1 [88.5, 100]	90.2 [81.0, 99.3]
	2019–2022	63*	96.8	100	97.2 [91.7, 100]	93.9 [85.8, 100]
Xc. Malignant gonadal germ cell tumours	1999–2003	45	95.6	97.8 [93.5, 100]	93.3 [86.1, 100]	93.3 [86.1, 100]
	2004–2008	66	89.4	100	95.3 [90.1, 100]	95.3 [90.1, 100]
	2009–2013	65	86.2	100	98.4 [95.3, 100]	98.4 [95.3, 100]
	2014–2018	72	95.8	98.6 [95.9, 100]	97.2 [93.3, 100]	97.2 [93.3, 100]
	2019–2022	113*	95.6	100	100	100

* The number of contributing cases for period survival are the number of patients diagnosed in 2014–2021 with survival experience during 2019–2022 up to 5 years after diagnosis.

Table S7. Survival results by incidence cohort for sarcomas and epithelial tumours. OS rates for 2019–2022 were estimated following the period approach.

Diagnostic group	Incidence cohort	No.	5y FU (%)	1y OS (%) [95% CI]	3y OS (%) [95% CI]	5y OS (%) [95% CI]
**VIII. Malignant bone tumours**	**1999–2003**	**239**	**97.9**	**92.8 [89.6, 96.1]**	**75.9 [70.5, 81.4]**	**70.8 [65.0, 76.6]**
	**2004–2008**	**270**	**96.3**	**91.8 [88.5, 95.1]**	**69.2 [63.7, 74.8]**	**62.0 [56.1, 67.8]**
	**2009–2013**	**295**	**98.3**	**93.6 [90.8, 96.4]**	**77.2 [72.4, 82.0]**	**71.0 [65.8, 76.2]**
	**2014–2018**	**325**	**98.5**	**92.6 [89.8, 95.5]**	**74.4 [69.6, 79.1]**	**67.9 [62.8, 72.9]**
	**2019–2022**	**460**	**98.5**	**94.8 [91.9, 97.7]**	**77.4 [72.2, 82.5]**	**69.1 [63.4, 74.8]**
VIIIa. Osteosarcomas	1999–2003	105	97.1	93.3 [88.4, 98.1]	79.7 [71.9, 87.4]	72.8 [64.2, 81.4]
	2004–2008	114	93.9	89.4 [83.7, 95.1]	69.4 [60.8, 78.0]	63.0 [53.9, 72.0]
	2009–2013	126	99.2	91.3 [86.3, 96.2]	79.4 [72.3, 86.4]	69.8 [61.7, 77.8]
	2014–2018	147	98.6	88.4 [83.3, 93.6]	70.6 [63.2, 78.]	65.8 [58.1, 73.5]
	2019–2022	195	99.0	91.8 [86.4, 97.3]	76.1 [68.1, 84.1]	69.0 [60.4, 77.7]
VIIIc. Ewing tumour and related sarcomas of bone	1999–2003	129	99.2	92.3 [87.6, 96.9]	72.1 [64.4, 79.8]	68.2 [60.2, 76.3]
	2004–2008	150	98.0	94.7 [91.1, 98.3]	69.9 [62.5, 77.2]	61.8 [54.0, 69.6]
	2009–2013	160	98.1	95.0 [91.6, 98.4]	74.3 [67.5, 81.1]	70.5 [63.4, 77.6]
	2014–2018	162	98.8	96.3 [93.4, 99.2]	75.9 [69.3, 82.5]	67.2 [60.0, 74.5]
	2019–2022	238	98.3	96.6 [93.4, 99.9]	76.1 [68.8, 83.5]	66.2 [58.1, 74.3]
**IX. Soft tissue and other extraosseous sarcomas**	**1999–2003**	**218**	**98.6**	**85.3 [80.6, 90.0]**	**70.5 [64.4, 76.6]**	**64.0 [57.6, 70.4]**
	**2004–2008**	**258**	**96.5**	**90.3 [86.7, 93.9]**	**77.4 [72.2, 82.5]**	**72.6 [67.1, 78.1]**
	**2009–2013**	**303**	**98.0**	**89.1 [85.6, 92.6]**	**74.8 [69.9, 79.7]**	**69.0 [63.8, 74.3]**
	**2014–2018**	**312**	**98.1**	**90.0 [86.7, 93.4]**	**78.8 [74.2, 83.3]**	**75.9 [71.1, 80.6]**
	**2019–2022**	**444**	**98.2**	**91.1 [87.3, 94.9]**	**80.8 [75.8, 85.9]**	**77.5 [72.2, 82.8]**
IXa. Rhabdomyo-sarcomas	1999–2003	126	98.4	88.1 [82.4, 93.8]	73.0 [65.2, 80.7]	66.5 [58.2, 74.8]
	2004–2008	141	97.2	90.1 [85.1, 95.0]	72.9 [65.6, 80.3]	67.9 [60.1, 75.6]
	2009–2013	157	98.7	92.3 [88.2, 96.5]	73.1 [66.2, 80.1]	67.3 [60.0, 74.7]
	2014–2018	147	98.6	94.6 [90.9, 98.2]	81.6 [75.4, 87.9]	77.5 [70.8, 84.3]
	2019–2022	219	98.2	93.3 [88.6, 98.1]	84.2 [77.5, 90.9]	78.8 [71.3, 86.4]
IXb, d, e. Non-rhabdomyo-sarcoma soft tissue sarcomas	1999–2003	92	98.9	81.4 [73.4, 89.4]	67.1 [57.4, 76.7]	60.5 [50.4, 70.5]
	2004–2008	117	95.7	90.5 [85.2, 95.9]	82.8 [75.9, 89.6]	78.3 [70.8, 85.9]
	2009–2013	145	97.2	85.4 [79.7, 91.2]	76.4 [69.5, 83.3]	70.7 [63.2, 78.1]
	2014–2018	162	97.5	85.7 [80.3, 91.1]	75.8 [69.2, 82.4]	73.9 [67.1, 80.7]
	2019–2022	222	98.2	88.8 [82.9, 94.8]	77.4 [69.9, 84.9]	75.8 [68.1, 83.4]
**XI. Epithelial neoplasms and melanomas**	**1999–2003**	**57**	**87.7**	**98.3 [94.8, 100]**	**98.3 [94.8, 100]**	**98.3 [94.8, 100]**
	**2004–2008**	**100**	**84.0**	**94.9 [90.6, 99.3]**	**92.9 [87.8, 98.0]**	**90.6 [84.8, 96.5]**
	**2009–2013**	**119**	**77.3**	**93.8 [89.4, 98.3]**	**89.2 [83.4, 95.0]**	**88.1 [82.0, 94.2]**
	**2014–2018**	**143**	**89.5**	**95.1 [91.6, 98.6]**	**93.7 [89.7, 97.7]**	**90.7 [85.9, 95.5]**
	**2019–2022**	**226**	**90.3**	**96.0 [92.2, 99.8]**	**94.2 [89.7, 98.7]**	**89.8 [84.1, 95.5]**
XIb. Thyroid carcinomas	1999–2003	22	90.9	100	100	100
	2004–2008	30	86.7	100	100	100
	2009–2013	32	81.3	100	100	100
	2014–2018	46	91.3	100	100	100
	2019–2022	74	91.9	100	100	100

* The number of contributing cases for period survival are the number of patients diagnosed in 2014–2021 with survival experience during 2019–2022 up to 5 years after diagnosis.

Table S8. Number of registered cases by age group in the 2009–2018 incidence cohort by diagnostic group.

Diagnostic group	No. cases (%)				
	<1 year	1–4 years	5–9 years	10–14 years	Total
**I-XII. All tumours**	**1,084 (10,8)**	**3,429 (34,1)**	**2,867 (28,5)**	**2,677 (26,6)**	**10,057**
**I-XII. All tumours, malignant**	**1,029 (11,2)**	**3,203 (34,7)**	**2,552 (27,7)**	**2,439 (26,4)**	**9,223**
I. Leukaemias	154 (5,5)	1,239 (44)	817 (29)	606 (21,5)	2,816
Ia. Lymphoid leukaemias	77 (3,4)	1,085 (47,6)	676 (29,6)	443 (19,4)	2,281
Ib. Acute myeloid leukaemias	63 (14,1)	137 (30,7)	117 (26,2)	129 (28,9)	446
II. Lymphomas	9 (0,8)	176 (15,2)	417 (36,1)	554 (47,9)	1,156
IIa. Hodgkin lymphomas	1 (0,2)	13 (3)	115 (26,4)	306 (70,3)	435
IIb. Non-Hodgkin lymphomas (except Burkitt)	7 (1,8)	77 (20,3)	143 (37,6)	153 (40,3)	380
IIc. Burkitt lymphoma	1 (0,3)	86 (25,3)	159 (46,8)	94 (27,6)	340
III. CNS	164 (6,7)	831 (33,9)	890 (36,3)	565 (23,1)	2,450
III. CNS, malignant	109 (6,7)	606 (37,3)	580 (35,7)	328 (20,2)	1,623
III. CNS, non-malignant	55 (6,7)	225 (27,2)	310 (37,5)	237 (28,7)	827
IIIa. Ependymomas and choroid plexus tumour	33 (12,4)	117 (44)	63 (23,7)	53 (19,9)	266
IIIa. Ependymomas and choroid plexus tumour, malignant	22 (10,5)	105 (50)	51 (24,3)	32 (15,2)	210
IIIb. Astrocytomas	53 (5,3)	345 (34,8)	377 (38)	216 (21,8)	991
IIIb. Astrocytomas, malignant	33 (6,1)	204 (37,6)	195 (35,9)	111 (20,4)	543
IIIb. Astrocytomas, non-malignant	20 (4,5)	141 (31,5)	182 (40,6)	105 (23,4)	448
IIIc. Intracranial and intraspinal embryonal tumours	40 (8)	196 (39)	184 (36,6)	83 (16,5)	503
IIId. Other gliomas	11 (3,4)	84 (25,6)	137 (41,8)	96 (29,3)	328
IV. Peripheral nervous cell tumours	367 (44,8)	358 (43,7)	72 (8,8)	22 (2,7)	819
IVa. Neuroblastoma and ganglioneuroblastoma	366 (45,2)	356 (44)	70 (8,7)	17 (2,1)	809
V. Retinoblastoma	125 (41,5)	166 (55,1)	9 (3)	1 (0,3)	301
VI. Renal tumours	86 (16,5)	285 (54,6)	128 (24,5)	23 (4,4)	522
VIa. Nephroblastoma	86 (16,9)	283 (55,7)	122 (24)	17 (3,3)	508
VII. Hepatic tumours	43 (30,3)	64 (45,1)	18 (12,7)	17 (12)	142
VIIa. Hepatoblastoma	41 (32,5)	61 (48,4)	16 (12,7)	8 (6,3)	126
VIII. Malignant bone tumours	1 (0,2)	39 (6,3)	189 (30,5)	391 (63,1)	620
VIIIa. Osteosarcomas	0 (0)	7 (2,6)	77 (28,2)	189 (69,2)	273
VIIIc. Ewing tumour and related sarcomas of bone	1 (0,3)	29 (9)	103 (32)	189 (58,7)	322
IX. Soft tissue sarcomas and other extraosseous sarcomas	67 (10,9)	179 (29,1)	162 (26,3)	207 (33,7)	615
IXa. Rhabdomyosarcomas	20 (6,6)	126 (41,4)	91 (29,9)	67 (22)	304
IXb+d+e. Non-rhabdomyosarcoma soft tissue sarcomas	47 (15,3)	50 (16,3)	71 (23,1)	139 (45,3)	307
X. Germ cell tumours	58 (17,4)	62 (18,6)	84 (25,2)	129 (38,7)	333
Xa. Intracranial and intraspinal germ cell tumours	5 (4,5)	7 (6,3)	47 (42)	53 (47,3)	112
Xb. Extracranial and extragonadal germ cell tumours	37 (46,8)	29 (36,7)	8 (10,1)	5 (6,3)	79
Xc. Gonadal germ cell tumours	16 (11,7)	25 (18,2)	29 (21,2)	67 (48,9)	137
XI. Epithelial neoplasms and melanomas	4 (1,5)	20 (7,6)	81 (30,9)	157 (59,9)	262
XIb. Thyroid carcinomas	0 (0)	2 (2,6)	26 (33,3)	50 (64,1)	78

Table S9. Observed survival at 5 years by sex and diagnostic group in the 2009–2018 incidence cohort, 0–14 years.

Diangostic group	Females		Males		P log-rank
	No.	5y Survival (95% CI)	No.	5y Survival (95% CI)	
**I-XII. All tumours**	**4,561**	**81.2 [80.1, 82.4]**	**5,496**	**82.3 [81.3, 83.3]**	**0.113**
**I-XII. All tumours, malignant**	**4,159**	**79.8 [78.6, 81.0]**	**5,064**	**81.0 [80.0, 82.1]**	**0.082**
I. Leukaemias	1,265	83.9 [81.9, 85.9]	1,551	84.9 [83.1, 86.7]	0.377
Ia. Lymphoid leukaemias	1,016	86.7 [84.6, 88.8]	1,265	87.2 [85.4, 89.1]	0.624
Ib. Acute myeloid leukaemias	208	71.0 [64.8, 77.2]	238	71.7 [65.9, 77.5]	0.710
II. Lymphomas	368	92.1 [89.3, 94.8]	788	94.2 [92.6, 95.9]	0.162
IIa. Hodgkin lymphomas	186	97.3 [94.9, 99.6]	249	97.6 [95.6, 99.5]	0.861
IIb. Non-Hodgkin lymphomas (except Burkitt)	116	81.9 [74.9, 88.9]	264	90.5 [86.9, 94.0]	0.016
IIc. Burkitt lymphoma	66	95.4 [90.2, 100]	274	94.9 [92.2, 97.5]	0.845
III. CNS	1,178	73.9 [71.3, 76.4]	1272	72.9 [70.5, 75.4]	0.772
III. CNS, malignant	779	62.5 [59.1, 65.9]	844	60.9 [57.6, 64.2]	0.707
III. CNS, non-malignant	399	96.4 [94.6, 98.3]	428	96.9 [95.3, 98.6]	0.677
IIIa. Ependymomas and choroid plexus tumour	130	71.5 [63.7, 79.2]	136	73.4 [65.9, 80.8]	0.593
IIIa. Ependymomas and choroid plexus tumour, malignant	106	65.9 [56.9, 75.0]	104	66.1 [57.0, 75.3]	0.824
IIIb. Astrocytomas	509	86.4 [83.4, 89.4]	482	83.5 [80.2, 86.9]	0.215
IIIb. Astrocytomas, malignant	280	75.9 [70.9, 80.9]	263	71.1 [65.6, 76.5]	0.198
IIIb. Astrocytomas, non-malignant	229	99.1 [97.9, 100]	219	98.6 [97.1, 100]	0.616
IIIc. Intracranial and intraspinal embryonal tumours	201	50.7 [43.8, 57.6]	302	51.2 [45.5, 56.8]	0.577
IIId. Other gliomas	174	55.9 [48.5, 63.3]	154	58.9 [51.1, 66.7]	0.635
IV. Peripheral nervous cell tumours	392	79.2 [75.1, 83.2]	427	78.7 [74.8, 82.6]	0.953
IVa. Neuroblastoma and ganglioneuroblastoma	388	79.0 [74.9, 83.0]	421	78.4 [74.5, 82.4]	0.931
V. Retinoblastoma	145	96.5 [93.4, 99.5]	156	98.1 [95.9, 100]	0.410
VI. Renal tumours	269	92.1 [88.9, 95.4]	253	93.7 [90.7, 96.7]	0.477
VIa. Nephroblastoma	264	92.0 [88.7, 95.3]	244	93.4 [90.3, 96.5]	0.510
VII. Hepatic tumours	61	83.5 [74.1, 92.8]	81	78.4 [69.3, 87.5]	0.528
VIIa. Hepatoblastoma	52	86.5 [77.2, 95.8]	74	79.1 [69.7, 88.5]	0.352
VIII. Malignant bone tumours	262	67.5 [61.8, 73.1]	358	70.8 [66.0, 75.5]	0.365
VIIIa. Osteosarcomas	129	62.7 [54.3, 71.1]	144	72.0 [64.7, 79.4]	0.129
VIIIc. Ewing tumour and related sarcomas of bone	128	71.1 [63.2, 78.9]	194	67.3 [60.7, 74.0]	0.561
IX. Soft tissue sarcomas and other extraosseous sarcomas	274	69.2 [63.7, 74.7]	341	75.2 [70.6, 79.8]	0.094
IXa. Rhabdomyosarcomas	123	67.5 [59.2, 75.8]	181	75.5 [69.3, 81.8]	0.103
IXb+d+e. Non-rhabdomyosarcoma soft tissue sarcomas	149	70.2 [62.8, 77.6]	158	74.5 [67.6, 81.3]	0.447
X. Germ cell tumours	187	92.4 [88.5, 96.2]	146	90.2 [85.4, 95.1]	0.494
Xa. Intracranial and intraspinal germ cell tumours	43	93.0 [85.4, 100]	69	82.3 [73.3, 91.4]	0.189
Xb. Extracranial and extragonadal germ cell tumours	52	86.2 [76.7, 95.7]	27	92.6 [82.7, 100]	0.441
Xc. Gonadal germ cell tumours	87	96.4 [92.5, 100]	50	100	0.186
XI. Epithelial neoplasms and melanomas	149	92.2 [87.8, 96.6]	113	86.0 [79.4, 92.6]	0.112
XIb. Thyroid carcinomas	53	100	25	100	-

Table S10. Age-standardised overall survival rates (StOS) by diagnostic group and incidence cohort. Age-standardisation was based on the 0–4, 5–9 and 10–14 years age groups unless otherwise stated (see footnotes).

Diagnostic group	1999–2003		2004–2008		2009–2013		2014–2018		2019–2022*	
	**n**	**5-year StOS [95% CI]**	**n**	**5-year StOS [95% CI]**	**n**	**5-year StOS [95% CI]**	**n**	**5-year StOS [95% CI]**	**n**	**5-year StOS [95% CI]**
**I-XII. All tumours**	**3,236**	**75.4 [73.9, 76.9]**	**4,218**	**76.5 [75.3, 77.8]**	**4,959**	**80.0 [78.9, 81.2]**	**5,098**	**83.6 [82.6, 84.6]**	**7,473**	**84.6 [83.5, 85.8]**
**I-XII. All tumours, malignant**	**3,010**	**74.2 [72.6, 75.7]**	**3,910**	**76.0 [74.6, 77.3]**	**4,565**	**78.8 [77.7, 80.0]**	**4,658**	**82.1 [81.0, 83.2]**	**6,753**	**83.1 [81.9, 84.4]**
I. Leukaemias	822	75.8 [72.9, 78.7]	1,146	77.4 [75.0, 79.9]	1,388	82.0 [80.0, 84.0]	1,428	86.8 [85.1, 88.6]	2,094	86.6 [84.6, 88.7]
Ia. Lymphoid leukaemias	659	79.7 [76.6, 82.7]	895	82.6 [80.1, 85.1]	1,133	84.7 [82.6, 86.8]	1,148	89.3 [87.5, 91.1]	1,726	89.8 [87.8, 91.9]
Ib. Acute myeloid leukaemias	144	59.5 [51.5, 67.6]	222	59.4 [52.9, 65.9]	214	67.9 [61.6, 74.2]	232	75.1 [69.5, 80.8]	297	72.6 [65.7, 79.6]
II. Lymphomas	452	86.4 [83.2, 89.6]	506	87.7 [84.9, 90.6]	571	93.1 [91.1, 95.2]	585	94.0 [92.1, 95.9]	918	94.4 [92.3, 96.6]
IIa. Hodgkin lymphomas†	185	94.5 [91.2, 97.7]	183	93.9 [90.4, 97.4]	212	97.6 [95.7, 99.5]	223	97.3 [95.2, 99.4]	366	97.8 [95.8, 99.9]
IIb. Non-Hodgkin lymphomas (except Burkitt)	146	75.2 [68.0, 82.3]	162	78.6 [72.5, 84.8]	188	88.6 [84.1, 93.1]	192	87.3 [82.7, 91.9]	297	87.9 [82.6, 93.1]
IIc. Burkitt lymphoma	120	87.1 [81.2, 93.0]	161	89.6 [84.8, 94.4]	171	92.3 [88.3, 96.2]	169	97.6 [95.7, 99.5]	254	97.6 [94.9, 100]
III. CNS	679	66.6 [63.0, 70.1]	930	66.7 [63.6, 69.7]	1,200	71.2 [68.6, 73.7]	1,250	75.9 [73.5, 78.3]	1,797	78.1 [75.4, 80.7]
III. CNS, malignant	456	53.8 [49.3, 58.4]	627	58.7 [54.8, 62.6]	808	60.0 [56.6, 63.4]	815	63.8 [60.5, 67.1]	1087	66.2 [62.5, 70.0]
III. CNS, non-malignant	223	92.2 [88.7, 95.7]	303	82.9 [78.7, 87.2]	392	94.2 [91.9, 96.4]	435	99.1 [98.2, 99.9]	710	99.4 [98.5, 100]
IIIa. Ependymomas and choroid plexus tumour‡	88	61.8 [52.0, 71.5]	112	65.9 [57.8, 74.0]	130	70.2 [62.5, 77.9]	136	73.4 [65.9, 80.9]	201	78.1 [69.9, 86.3]
IIIa. Ependymomas and choroid plexus tumour, malignant‡	71	51.6 [40.7, 62.6]	96	60.2 [51.3, 69.1]	110	65.9 [57.1, 74.6]	100	65.3 [56.0, 74.6]	146	71.3 [60.8, 81.8]
IIIb. Astrocytomas	273	80.1 [75.4, 84.9]	326	78.3 [73.8, 82.8]	453	85.0 [81.7, 88.3]	538	84.6 [81.6, 87.7]	777	88.6 [85.5, 91.8]
IIIb. Astrocytomas, malignant	156	69.8 [62.6, 77.0]	188	70.8 [64.3, 77.2]	240	72.6 [67.0, 78.1]	303	72.7 [67.8, 77.6]	389	77.1 [71.2, 83.0]
IIIb. Astrocytomas, non-malignant	117	94.9 [91.3, 98.5]	138	90.1 [85.2, 94.9]	213	98.2 [96.6, 99.8]	235	99.6 [99, 100.2]	388	100
IIIc. Intracranial and intraspinal embryonal tumours	154	47.3 [39.8, 54.9]	216	54.0 [47.4, 60.6]	284	50.2 [44.4, 55.9]	219	52.7 [46.3, 59.2]	287	64.6 [57.3, 71.9]
IIId. Other gliomas	55	46.1 [33.4, 58.7]	115	49.9 [40.6, 59.2]	153	56.6 [49.2, 63.9]	175	60.6 [53.6, 67.7]	239	49.9 [42.0, 57.7]
IV. Peripheral nervous cell tumours‡	353	72.2 [67.6, 76.7]	402	74.0 [69.8, 78.2]	420	75.3 [71.2, 79.5]	399	82.4 [78.6, 86.1]	557	85.0 [80.8, 89.2]
IVa. Neuroblastoma and ganglioneuroblastoma‡	351	72.2 [67.7, 76.8]	398	74.0 [69.7, 78.2]	417	75.2 [71.1, 79.4]	392	81.9 [78.1, 85.8]	547	84.8 [80.4, 89.1]

Table S10 (continued)

Diagnostic group	1999–2003		2004–2008		2009–2013		2014–2018		2019–2022*	
	n	5-year StOS [95% CI]	n	5-year StOS [95% CI]	n	5-year StOS [95% CI]	n	5-year StOS [95% CI]	n	5-year StOS [95% CI]
V. Retinoblastoma§	100	96.9 [93.4, 100]	133	96.9 [93.9, 99.9]	152	95.4 [92.0, 98.7]	149	99.3 [98.0, 100]	218	99.0 [97.2, 100]
VI. Renal tumours‡	150	87.1 [81.6, 92.5]	252	88.7 [84.6, 92.7]	273	91.9 [88.7, 95.1]	249	94.2 [91.4, 97.1]	378	92.6 [88.9, 96.3]
VIa. Nephroblastoma‡	146	86.7 [81.2, 92.3]	248	88.5 [84.4, 92.6]	268	91.8 [88.5, 95.1]	240	94.0 [91.0, 96.9]	365	92.8 [89.1, 96.6]
VII. Hepatic tumours§	46	69.2 [55.8, 82.7]	65	75.1 [64.5, 85.7]	74	86.1 [78.1, 94.1]	68	74.8 [64.5, 85.2]	96	79.8 [68.6, 91.0]
VIIa. Hepatoblastoma§	39	74.4 [60.7, 88.1]	59	79.7 [69.4, 89.9]	70	86.8 [78.8, 94.9]	56	76.6 [65.5, 87.7]	84	81.1 [69.3, 92.9]
VIII. Malignant bone tumours	239	70.5 [64.7, 76.3]	270	62.1 [56.3, 68.0]	295	70.9 [65.7, 76.1]	325	67.4 [62.3, 72.5]	460	69.6 [63.9, 75.2]
VIIIa. Osteosarcomas†	105	72.8 [64.1, 81.4]	114	62.6 [53.5, 71.7]	126	69.8 [61.7, 77.8]	147	65.6 [57.9, 73.4]	195	69.7 [61.2, 78.3]
VIIIc. Ewing tumour and related sarcomas of bone†	129	67.7 [59.6, 75.7]	150	61.5 [53.8, 69.2]	160	70.5 [63.5, 77.6]	162	66.3 [59.1, 73.6]	238	66.9 [59.0, 74.8]
IX. Soft tissue sarcomas and other extraosseous sarcomas	218	63.9 [57.5, 70.3]	258	72.7 [67.2, 78.2]	303	69.1 [63.9, 74.3]	312	76.1 [71.4, 80.9]	444	77.7 [72.4, 83.0]
IXa. Rhabdomyosarcomas	126	66.3 [57.9, 74.6]	141	67.7 [60.0, 75.4]	157	67.3 [60.0, 74.7]	147	78.0 [71.4, 84.7]	219	80.0 [73.0, 87.0]
IXb+d+e. Non-rhabdomyosarcoma soft tissue sarcomas	92	60.4 [50.3, 70.4]	117	78.9 [71.5, 86.3]	145	70.8 [63.4, 78.2]	162	74.0 [67.2, 80.8]	222	74.8 [67.0, 82.7]
X. Germ cell tumours	113	84.1 [77.4, 90.8]	150	85.6 [79.8, 91.3]	155	93.3 [89.4, 97.2]	178	89.7 [85.2, 94.2]	268	96.2 [93.0, 99.5]
Xa. Intracranial and intraspinal germ cell tumours†	37	78.5 [67.5, 89.5]	45	71.7 [58.5, 84.9]	48	93.4 [87.3, 99.6]	64	81.7 [72.7, 90.8]	83	92.7 [84.8, 100]
Xb. Extracranial and extragonadal germ cell tumours§	30	80.0 [65.7, 94.3]	38	86.5 [75.4, 97.5]	38	86.7 [75.8, 97.6]	41	90.2 [81.0, 99.3]	63	93.9 [85.8, 100]
Xc. Gonadal germ cell tumours†	45	93.1 [86.6, 99.5]	66	95.4 [91.2, 99.6]	65	98.6 [96.5, 100	72	97.2 [94.3, 100]	113	100
XI. Epithelial neoplasms and melanomas†	57	98.4 [96.0, 100]	100	90.8 [85.2, 96.3]	119	88.2 [82.1, 94.3]	143	90.5 [85.7, 95.3]	226	89.5 [83.6, 95.3]
XIb. Thyroid carcinomas§	22	100	30	100	32	100	46	100	74	100

* Survival estimates for 2019–2022 were obtained using the period approach with cases diagnosed during 2014–2021. The column 'n' reflects the number of contributing cases.

† Age-standardisation was performed using 0–9 and 10–14 years age groups.

‡ Age-standardisation was performed using 0–4 and 5–14 years age groups.

§ Age-standardisation was not performed. Observed 0–14 years estimates are provided.

Table S11. Number of recorded epithelial tumour cases by incidence cohort, 0–14 years.

**Diagnostic subgroup or division**	**Incidence cohort**					**Total**
	**1999–2003**	**2004–2008**	**2009–2013**	**2014–2018**	**2019–2021**	**(1999–2021)**
XIa. Adrenocortical carcinomas	1	13	8	4	9	**35**
XIb. Thyroid carcinomas	22	30	32	46	28	**158**
XIc. Nasopharyngeal carcinomas	7	3	7	10	7	**34**
XId. Malignant melanomas	9	18	20	15	13	**75**
XIe. Skin carcinomas	4	2	1	8	0	**15**
XIf. Other and unspecified carcinomas	14	34	51	60	34	**193**
XIf1. Carcinomas of salivary glands	2	9	4	9	9	**33**
XIf2. Carcinomas of colon and rectum	1	0	6	3	1	**11**
XIf3. Carcinomas of appendix	3	13	23	27	15	**81**
XIf4. Carcinomas of lung	2	5	2	8	2	**19**
XIf5. Carcinomas of thymus	0	1	2	2	2	**7**
XIf6. Carcinomas of breast	0	0	0	0	0	**0**
XIf7. Carcinomas of cervix uteri	0	0	0	0	0	**0**
XIf8. Carcinomas of bladder	2	3	0	1	1	**7**
XIf9. Carcinomas of eye	0	0	2	0	0	**2**
XIf10. Carcinomas of other specified sites	3	2	11	9	2	**27**
XIf11. Carcinomas of unspecified site	1	1	1	1	2	**6**
**XI. Epithelial neoplasms and melanomas**	**57**	**100**	**119**	**143**	**91**	**510**
